# A Prognostic Model of Non-Small Cell Lung Cancer With a Radiomics Nomogram in an Eastern Chinese Population

**DOI:** 10.3389/fonc.2022.816766

**Published:** 2022-06-14

**Authors:** Lijie Wang, Ailing Liu, Zhiheng Wang, Ning Xu, Dandan Zhou, Tao Qu, Guiyuan Liu, Jingtao Wang, Fujun Yang, Xiaolei Guo, Weiwei Chi, Fuzhong Xue

**Affiliations:** ^1^ Department of Epidemiology and Health Statistics, School of Public Health, Cheeloo College of Medicine, Shandong University, Jinan, China; ^2^ Department of Pulmonary and Critical Care Medicine, Weihai Municipal Hospital, Cheeloo College of Medicine, Shandong University, Weihai, China; ^3^ Shandong Provincial Key Laboratory of Immunohematology, Qilu Hospital, Cheeloo College of Medicine, Shandong University, Jinan, China; ^4^ Department of Radiology, Weihai Municipal Hospital, Cheeloo College of Medicine, Shandong University, Weihai, China; ^5^ Department of Hematology, Qilu Hospital, Cheeloo College of Medicine, Shandong University, Jinan, China; ^6^ Department of Oncology, Weihai Municipal Hospital, Cheeloo College of Medicine, Shandong University, Weihai, China; ^7^ The Department for Chronic and Non-Communicable Disease Control and Prevention, Shandong Center for Disease Control and Prevention, Jinan, China; ^8^ National Administration of Health Data, Jinan, China; ^9^ Institute for Medical Dataology, Shandong University, Jinan, China

**Keywords:** non-small cell lung cancer, computed tomography, radiomics, nomogram, survival, TNM staging

## Abstract

**Background:**

The aim of this study was to build and validate a radiomics nomogram by integrating the radiomics features extracted from the CT images and known clinical variables (TNM staging, etc.) to individually predict the overall survival (OS) of patients with non-small cell lung cancer (NSCLC).

**Methods:**

A total of 1,480 patients with clinical data and pretreatment CT images during January 2013 and May 2018 were enrolled in this study. We randomly assigned the patients into training (*N* = 1036) and validation cohorts (*N* = 444). We extracted 1,288 quantitative features from the CT images of each patient. The Least Absolute Shrinkage and Selection Operator (LASSO) Cox regression model was applied in feature selection and radiomics signature building. The radiomics nomogram used for the prognosis prediction was built by combining the radiomics signature and clinical variables that were derived from clinical data. Calibration ability and discrimination ability were analyzed in both training and validation cohorts.

**Results:**

Eleven radiomics features were selected by LASSO Cox regression derived from CT images, and the radiomics signature was built in the training cohort. The radiomics signature was significantly associated with NSCLC patients’ OS (HR = 3.913, *p* < 0.01). The radiomics nomogram combining the radiomics signature with six clinical variables (age, sex, chronic obstructive pulmonary disease, T stage, N stage, and M stage) had a better prognostic performance than the clinical nomogram both in the training cohort (C-index, 0.861, 95% CI: 0.843–0.879 vs. C-index, 0.851, 95% CI: 0.832–0.870; *p* < 0.001) and in the validation cohort (C-index, 0.868, 95% CI: 0.841–0.896 vs. C-index, 0.854, 95% CI: 0.824–0.884; *p* = 0.002). The calibration curves demonstrated optimal alignment between the prediction and actual observation.

**Conclusion:**

The established radiomics nomogram could act as a noninvasive prediction tool for individualized survival prognosis estimation in patients with NSCLC. The radiomics signature derived from CT images may help clinicians in decision-making and hold promise to be adopted in the patient care setting as well as the clinical trial setting.

## 1 Introduction

Lung cancer is one of the most common types of cancer and a major cause of mortality worldwide for both men and women (1). Non-small cell lung cancer (NSCLC) is the most common type, which constitutes 84% of all lung cancer cases (2). Moreover, the 5-year survival rate following the diagnosis for the patients is as low as 17%, even though the prognosis and treatment of lung cancer have already been notably improved (3).

The tumor, node, and metastasis (TNM) staging is the most commonly used and universally accepted staging (clinical and pathological) system for cancer. TNM staging is simple to apply and can be highly discriminatory for survival (4). TNM staging is also beneficial in defining optimal therapeutic strategies in clinical trials. TNM staging gives an indication of prognosis as a probability of survival, but not identifying individual outcomes (5). Current prediction models are based on clinical, imaging, and/or pathological information. We can and should look to improve our classifications by determining additional effective prognostic indicators to achieve individualized management in clinical practice.

Recently, some studies have demonstrated the relations between lung cancer-related genes of tumors and survival prognosis, which can be used to improve the predictions from the traditional TNM staging strategies (6). The direct application of such early genetic information without clinical validation is clinically and ethically concerning (7). Tumors are spatially heterogeneous, making it difficult to apply biopsy data in a meaningful way.

Nowadays, with the help of non-invasive techniques and the ability to extract high-precision information, medical imaging becomes a clinical routine for diagnosis and prognosis (8). As an emerging methodology, radiomics has been used to analyze complicated and confounding information and then quantitatively extract valuable information from medical images by using high-throughput calculations (9). To be more specific, radiomics converted the images into mineable, comprehensive quantitative features following four steps (1): image acquisition and reconstruction, (2) segmentation of ROI, (3) feature extraction and quantification, and (4) model construction (10). The radiomics features can be extracted from not only unmanipulated medical images but also images processed by Gaussian and wavelet filters (8).

In the case of lung cancer, medical images reflecting features of tumors usually rely on radiological data (e.g., chest CT and brain MRI scans). At present, it is a common clinical practice for lung cancer patients to undergo CT examinations in order to identify tumor size and location. Features from CT can be used to predict the malignant potential of a nodule on a chest CT based on the correlations between them (11, 12). In addition, some features of a nodule are identified to be closely related to diagnosis (e.g., lung cancer screening) and tumor genomics (13). In the last decade, many studies have been conducted to figure out which factors measured from CT can be correlated to overall survival (OS) for the tumor patients. Radiomics can also be applied to predict response to some certain treatments (14).

Some previous studies have investigated the correlation between radiomics features and survival (15–20). Hawkins et al. built the classifiers that could predict survival time for adenocarcinoma using CT image features. The highest classification accuracy (AUC) was 77.5% (15). Yang et al. showed that PET/CT imaging data can be potentially used as a biomarker combined with clinical factors (distant metastasis, carcinoembryonic antigen, stage, and targeted therapy) in risk stratification for the OS with NSCLC patients. The performance of the model was 0.789 measured by the Harrell’s concordance statistic (C-index) (16). The C-index was the most commonly used performance measure to evaluate the discriminative ability of the developed models for survival data. The calculation of C-index considered the situation of censoring by interpreting for a pair of patients with and without the outcome. It ranged between 0.5 and 1.0; 0.5 indicated the random guesses and 1.0 represented that the predicted probabilities were perfectly the same as the observed survival information. A C-statistic of 0.7 to 0.8 was acceptable, while a C-statistic greater than 0.8 indicated good performance (21, 22). Botta et al. developed the model based on radiomic and clinical features (tumor location and T stage) for OS prediction, and had moderate performance with C-index = 0.57 (17). Xu et al. demonstrated that integrating CT scans at several different time points by the deep learning method could improve clinical prognosis predictions of patients with locally advanced NSCLC (e.g., 2-year OS: AUC = 0.74, *p* < 0.05) (18). Khorrami et al. showed that changes in CT radiographic characteristics correlated with lymphocyte distribution and could predict OS (HR: 1.64, 95% CI: 1.22–2.21, *p* = 0.001, C-index = 0.72) and response to immunotherapy in NSCLC (19). Yang et al. developed a radiomics nomogram by integrating the radiomics signatures extracted from combined 2D and 3D CT images and clinical factors (age, sex, T stage, and N stage) to evaluate the OS with NSCLC patients (C-index = 0.710) (20). However, these studies used limited mining of imaging data due to relatively small sample sizes or only a small number of extracted radiomics features.

Nomograms are generally accepted as a useful and reliable tool to evaluate risk and predict individualized cancer prognosis (23). Our team had successfully developed a radiomics nomogram to distinguish malignant from benign pulmonary nodules for the early screening and diagnosis of lung cancer clinically (24). In this study, we aimed to further develop a radiomics nomogram incorporating traditional clinical factors, such as TNM, for predicting the OS of patients with NSCLC.

## 2 Materials and Methods

### 2.1 Study Cohort

A total of 1,524 patients with clinical data and pretreatment chest CT images at the Weihai Municipal Hospital during January 2013 and May 2018 were identified in this study. The inclusion criteria were defined as follows: (1) patients were histopathologically confirmed with NSCLC either by surgical specimen or by preoperative biopsy, (2) patients who received non-contrast-enhanced CT scans during the diagnosis, (3) patients aged ≥18 years, and (4) patients not diagnosed with lung cancer or other types of malignant tumors in the past 5 years. The exclusion criteria were defined as follows: (1) CT images were too blurry to identify the patient’s tumor area, and (2) eligible variables were incomplete. The study protocol was conducted under approval by the Public Health Ethics Committee of Shandong University (Approval No. 20180801). The requirement for informed consent was waived because of the retrospective nature of the study.

### 2.2 Ascertainment of Exposures

The exposure information consisted of two parts: the clinical data and the assessment of CT scans for each patient. In our study, clinical data were collected from electronic medical record and examination data by oncologists (AL, NX, and DZ) and a data manager (ZW). The following variables were collected: the sociodemographics of the patients (age at diagnosis, sex, smoking status, and drinking status), the presence of any comorbidity [chronic obstructive pulmonary disease (COPD), hypertension, diabetes, coronary heart disease (CHD), and cerebrovascular disease], and pathologic data [histopathological diagnosis, tumor location, tumor (T stage), node (N stage), metastasis status (M stage), and staging].

Smoking status was categorized as never, former, and current smoking. Drinking status was categorized as yes and no. Comorbidities were defined according to the International Classification of Diseases, 10th Revision (ICD-10) code: CODP, J40–J44; hypertension, I10–I15; diabetes, E10–E14; CHD, I20–I25; and cerebrovascular disease, I60–I69. The location of the tumor was categorized as the right upper, right middle, right lower, left upper, and left lower. Pathologic staging was defined in accordance with the eighth edition of the American Joint Committee on Cancer TNM staging system (25).

### 2.3 Image Acquisition and Radiomics Feature Extraction

The radiomics workflow is presented in **Supplementary Figure S1**. CT scans were obtained with participants at baseline before percutaneous puncture, bronchoscopic biopsy, or surgery. The included CT images were carried out using a SIEMENS SOMATOM Definition Flash system (Siemens Healthineers, Erlangen, Germany). Detailed CT scan parameters for the reconstructed image were described as follows: the tube voltage, 120 kV; current, 150 effective mAs; beam collimation, 128 × 0.6 mm; pitch, 1.2; slice thickness, 1.0 mm; and gantry rotation time, 0.5 s. These settings were the same for all patients in our study. Both lung window and mediastinum window were included in the CT images of each patient. Here, we used the lung window of the CT images to extract radiomics features.

The segmentation of the tumor region of interest was performed using 3D Slicer (www.slicer.org), a free open-source software (version 4.8.0, National Institutes of Health, Bethesda, MD, USA) (26). Each CT image was semi-automatically segmented for each lesion slice by slice by two doctors in Weihai Municipal Hospital, Weihai, Shandong, China (Dr. Ailing Liu, Department of Respiratory Internal Medicine; Dr. Guiyuan Liu, Department of Radiology) who were blinded to the patient cohort. The guidance of tumor segmentation is listed in Supplementary Material. The interobserver variability of radiomics feature extraction was estimated by the intraclass correlation coefficients (ICC) (27). An ICC value greater than 0.75 was considered to represent good agreement.

The CT scans acquired in the clinical processes, as well as those processed with Gaussian and wavelet filters, had their quantitative radiomics features extracted by using the PyRadiomics library (version 2.1), a free open-source python (version 3.6, https://www.python.org/) package that provides many options to customize extracting the radiomics features from CT images (28). The calculation methods for each radiomics feature were described in the following website: https://pyradiomics.readthedocs.io/en/latest/features.html. Laplacians of Gaussian filtering or wavelet filtering were used in image pre-processing. All radiomics features were defined, adhered to the Imaging Biomarkers Standardization Initiative guidelines, and were assigned into the following three groups: (1) first-order features, (2) shape features, and (3) texture features (29). The features were extracted from the original images and the pre-processed images. In total, 1,288 radiomics features were extracted from CT images per patient (Supplementary Material and Supplementary **Table 1**). More detailed information about the process of image acquisition and reconstruction, region of interest segmentation, and feature extraction and quantification has previously been described by Liu et al. (24).

**Table 1:** Baseline demographic and clinical characteristics in the study.

Variable	Level	Training data (*N* = 1,036)	Validation data (*N* = 444)	Total (*N* = 1,480)	*p*-value
Age	Mean (SD) median	60.63 (8.77) 61.00	61.08 (8.85) 61.00	60.77 (8.79) 61.00	0.360
Sex	Female	517 (49.90)	216 (48.65)	733 (49.53)	0.700
	Male	519 (50.10)	228 (51.35)	747 (50.47)	
Tumor location	Right upper	321 (30.98)	140 (31.53)	461 (31.15)	0.958
	Right lower	196 (18.92)	89 (20.05)	285 (19.26)	
	Right middle	103 (9.94)	40 (9.01)	143 (9.66)	
	Left upper	236 (22.78)	97 (21.85)	333 (22.50)	
	Left lower	180 (17.37)	78 (17.57)	258 (17.43)	
Smoking status	Never	686 (66.22)	307 (69.14)	993 (67.09)	0.455
	Current	222 (21.43)	91 (20.50)	313 (21.15)	
	Former	128 (12.36)	46 (10.36)	174 (11.76)	
Drinking status	No	866 (83.59)	386 (86.94)	1,252 (84.59)	0.120
	Yes	170 (16.41)	58 (13.06)	228 (15.41)	
T stage	T1	517 (49.90)	242 (54.50)	759 (51.28)	0.131
	T2	338 (32.63)	141 (31.76)	479 (32.36)	
	T3	77 (7.43)	32 (7.21)	109 (7.36)	
	T4	104 (10.04)	29 (6.53)	133 (8.99)	
N stage	N0	708 (68.34)	324 (72.97)	1,032 (69.73)	0.218
	N1	92 (8.88)	32 (7.21)	124 (8.38)	
	N2	159 (15.35)	65 (14.64)	224 (15.14)	
	N3	77 (7.43)	23 (5.18)	100 (6.76)	
M stage	M0	921 (88.90)	385 (86.71)	1,306 (88.24)	0.267
	M1	115 (11.10)	59 (13.29)	174 (11.76)	
COPD	No	721 (69.59)	333 (75.00)	1,054 (71.22)	0.041
	Yes	315 (30.41)	111 (25.00)	426 (28.78)	
Hypertension	No	755 (72.88)	306 (68.92)	1,061 (71.69)	0.137
	Yes	281 (27.12)	138 (31.08)	419 (28.31)	
Diabetes	No	935 (90.25)	390 (87.84)	1,325 (89.53)	0.195
	Yes	101 (9.75)	54 (12.16)	155 (10.47)	
CHD	No	926 (89.38)	413 (93.02)	1,339 (90.47)	0.037
	Yes	110 (10.62)	31 (6.98)	141 (9.53)	
Cerebrovascular disease	No	1,010 (97.49)	432 (97.30)	1,442 (97.43)	0.971
	Yes	26 (2.51)	12 (2.70)	38 (2.57)	

SD, standard deviation; COPD, chronic obstructive pulmonary disease; CHD, coronary heart disease.

### 2.4 Follow-Up

The study outcome was OS, which was calculated from the date of diagnosis (date of surgery or biopsy) to the date of death, recorded *via* linkages to the database of death registries of Shandong Province by civil ID number, or May 30, 2021, whichever occurred first.

### 2.5 Statistical Analysis

**Figure 1:**
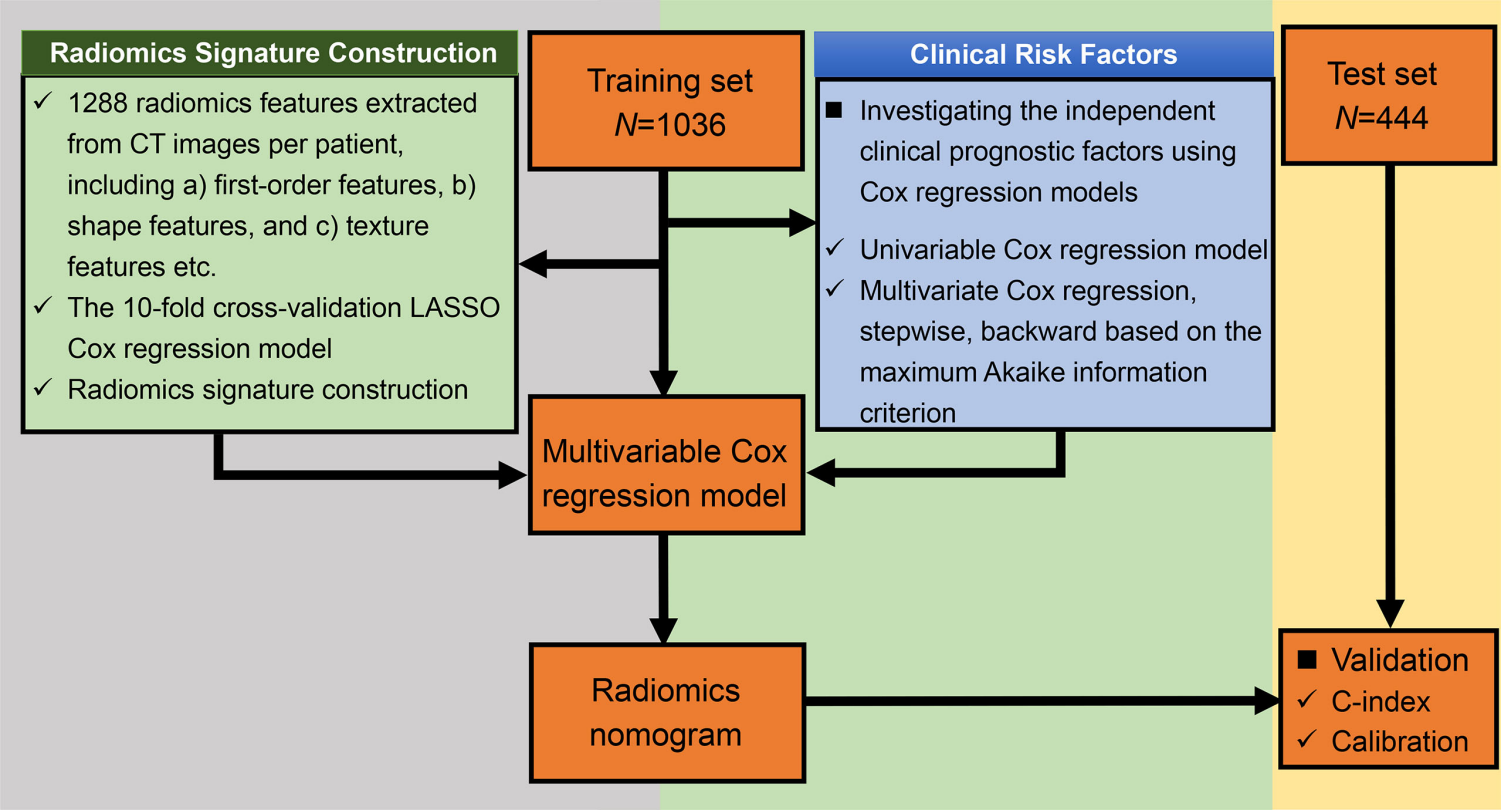
The flowchart of the study.

We randomly assigned 70% of the patients to the training cohort, and 30% to the validation cohort. The training cohort was used to develop the model while the validation cohort was used to qualify the performance of the model. **Figure 1** shows the flowchart of the study.

#### 2.5.1 Descriptive Analyses

Means ± standard deviations (SDs) or medians [interquartile ranges (IQRs)] were reported for quantitative variables. Frequencies and proportions (*N*, %) were reported for categorical variables. Quantitative baseline variables were compared with *t*-tests. Categorical variables were compared by performing the chi-square tests between groups.

#### 2.5.2 Construction of the Radiomics Signature

We used the 10-fold cross-validation Least Absolute Shrinkage and Selection Operator (LASSO) Cox regression model, which was an effective attractive method for high-dimensional data in survival analysis, to select the optimal nonzero coefficient features in the training cohort in order to reduce model overfitting (30). The most critical step for the LASSO Cox regression model was to determine the optimized hyperparameter λ, which ensured minimal model deviation. The radiomics signature for each participant was the weighted sum of all the selected radiomics features in terms of the following formula: $Radiomics\;signature\;=\;\sum_{i=1}^{N}coef_{i}X_{i}$ , where *N* is defined as the total number of selected feature, *coef*_*i*_ is the value of non-zero coefficient of the *i*th selected feature, and *X*_*i*_ is the value of the *i*th selected feature. The C-index was calculated so as to evaluate the predictive performance of the radiomics signature in both the training and validation cohorts (31). In addition, to validate the potential correlation between radiomics signature with OS, we categorized the patients as low-level and high-level risk based on their median radiomics signature. The Kaplan–Meier method was performed to estimate the OS of the two groups, while the difference in the survival curves was tested using the log-rank test.

#### 2.5.3 Construction and Validation of the Clinical Model

The correlation between OS and each clinical variable including the TNM stage was first analyzed with the univariable Cox regression model. Significant clinical variables whose *p*-value was less than 0.05 in the univariable analysis were evaluated using the Kaplan–Meier method and then were integrated into a multivariable Cox regression model to identify independent prognostic factors. The final multivariable Cox regression model was constructed using the stepwise backward variable selection process based on the maximum Akaike information criterion (AIC) (32). The corresponding C-index was calculated for evaluating the performance of the predictive probability of OS for each patient in both the training and the validation cohorts.

#### 2.5.4 Construction and Evaluation of a Radiomics Nomogram

Finally, the radiomics nomogram for predicting the OS was established based on the multivariable Cox regression model by combining the radiomics signatures and all the independent clinical risk factors derived from the clinical model. To test the robustness of the risk factors in the radiomics nomogram, we also did the stepwise Cox regression with all the risk factors (the clinical risk factors and the radiomics signatures) to see if the same risk factors remained in this time. The C-index and calibration curve were calculated to evaluate the validity of the established radiomics nomogram (33). To further validate the prognostic ability, the survival probabilities of all the patients were classified into four subgroups using the quartile values derived from the radiomics nomogram as thresholds. Survival curves were estimated for four subgroups using the Kaplan–Meier method and compared statistically using the log-rank test.

#### 2.5.5 Assessment of the Incremental Value of Radiomics Signature

The incremental value of the radiomics signatures to the clinical factors was evaluated by comparing the performance of the radiomics nomogram derived in this study with the clinical model in respect of discrimination (C-index) and calibration (calibration curves). The calibration curves were constructed to show accordance between nomogram-predicted survival probability with the observed survival probability using 1,000 bootstrap resamples (33). The calibration curve along the diagonal line indicated that the predicted probabilities were exactly the same as the actual outcomes, which is the hypothetical perfect situation.

All statistical analyses were performed using the R software (http://www.r-project.org) and a two-tailed *p*-value less than 0.05 was regarded as statistically significant. The LASSO Cox regression was performed using the R package *glmnet*. Stepwise AIC was implemented using the R function “step”, and the nomogram was built using the “rms” package.

## 3 Results

Among 1,524 patients, 44 patients (2.89%) were excluded because they were missing T stage (23 patients) and N stage (37 patients), respectively. Overall, there were 1,480 patients histologically confirmed with NSCLC with complete information in both clinical and CT image data in our study. All the 1,480 patients enrolled had complete follow-up information. Among them, adenocarcinoma (*N* = 1,218) and squamous cell carcinoma (*N* = 235) accounted for approximately 98% of patients, while other histology types, such as adenosquamous carcinoma and large cell lung cancer, were present in 2% of patients. Stage distribution of patients was listed as follows: stage IA = 666, stage IB = 220, stage IIA = 42, stage IIB = 125, stage IIIA = 167, stage IIIB = 71, stage IIIC = 15, and stage IV = 174. The observed numbers of deaths were 397 of 1,480 patients. Median follow-up time was 4.06 years (range, 9 days–8.02 years). The median age at diagnosis was 61 years. Approximately one-third of the patients had a history of smoking and COPD. We randomly divided the data into training (*N* = 1036) and validation cohorts (*N* = 444). The clinical characteristics are summarized in **Table 1**.

### 3.1 Feature Selection and Radiomics Signature Building

The interobserver ICCs ranged from 0.790 to 0.937, indicating favorable interobserver feature extraction reproducibility. Eleven features with non-zero coefficients were taken as the predictive radiomics features, which were obtained by the LASSO Cox regression model using 10-fold cross-validation in the training cohort (**Supplementary Materials** and **Supplementary Figure S2**). The optimal *λ* was 0.064 when the model had the minimum deviance. Then, an individual patient’s radiomics signature was calculated as a linear combination of the selected features weighted by their respective LASSO coefficients (**Supplementary Table 2**).

### 3.2 Prognostic Validation of the Radiomics Signature

Cox regression analyses showed that radiomics signatures were significantly associated with OS for NSCLC in both the training cohort (*p* < 0.001, HR = 3.913, 95% CI: 3.367–4.547) and the validation cohort (*p* < 0.001, HR = 3.867, 95% CI: 3.100–4.824). Additionally, the performance of radiomics signatures for predicting OS for NSCLC was evaluated using the Cox regression model. The radiomics signature yielded a C-index of 0.808 (95% CI: 0.784–0.831) on the training cohort and 0.820 (95% CI: 0.786–0.853) on the validation set (**Table 2**).

**Table 2:** The C-index with 95% confidence intervals calculated for the training and validation cohorts.

	Training cohort			Testing cohort	
	C-index	95% CI		C-index	95% CI
Radiomics signature	0.808	0.784–0.831		0.820	0.786–0.853
Clinical model	0.851	0.832–0.870		0.854	0.824–0.884
Radiomics nomogram	0.861	0.843–0.879		0.868	0.841–0.896

CI, confidence interval.

Furthermore, the patients were stratified into low-risk and high-risk groups in terms of the median value of the radiomics signature (−0.716). The Kaplan–Meier method was performed in the training and validation cohorts to analyze the association of the radiomics signature with OS in NSCLC patients (**Supplementary Figure S3**). Apparently, patients from the low-risk group had a notably better OS when compared with those in the high-risk group by the log-rank test in the training set (*p* < 0.001). The consensus result was found in the validation cohort.

### 3.3 Construction of Clinical Model and Radiomics Nomogram

Univariate Cox regression analyses showed that age, sex, smoking status, drinking status, COPD, T stage, N stage, and M stage were significantly associated with an increased risk of death for patients with NSCLC in the training cohort (all *p* < 0.01, **Supplementary Figure S4**). Kaplan–Meier survival analysis showed significant difference in the OS by each clinical factor (**Supplementary Figure S5**). Multivariable Cox analysis included these eight clinical variables and was performed using backward stepwise feature selection based on the maximum AIC. The final clinical Cox regression model included six clinical variables, namely, age, sex, COPD, T stage, N stage, and M stage (all *p* < 0.05, **Supplementary Figure S6).**

**Figure 2:**
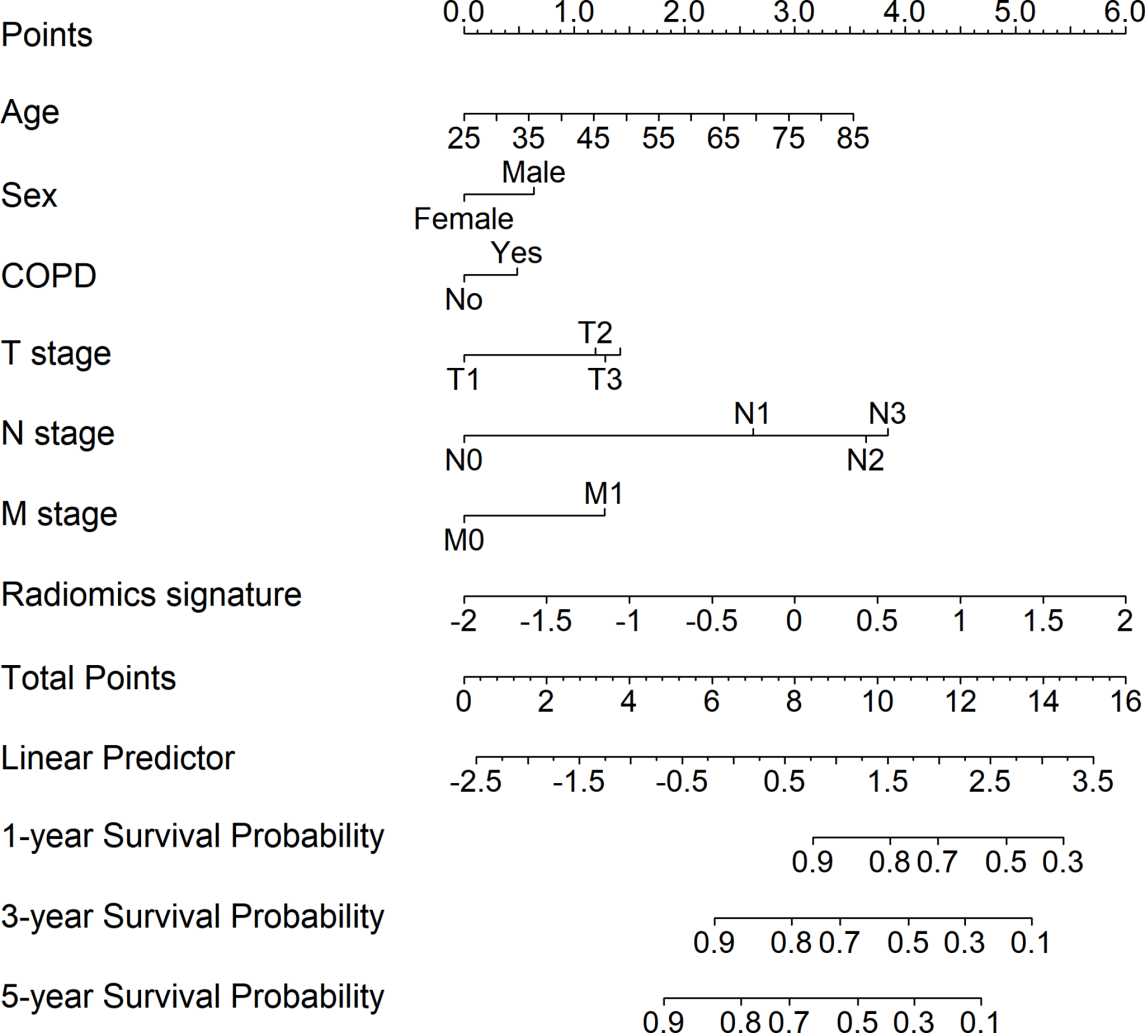
Development of the radiomics nomogram for patients with non-small cell lung cancer by integrating the radiomics signature with clinical information to predict the probability of overall survival at 1, 3, and 5 years.

We integrated the radiomics signatures with the six clinical variables to apply the stepwise multivariate Cox model, which identified that the radiomics signature remained an independent prognostic factor even after adjusting for clinical variables (*p* < 0.001, HR = 1.829, 95% CI: 1.465–2.283, **Supplementary Figure S7**). On the basis of the final multivariable Cox model, we constructed a radiomics nomogram that visually depicted the multivariate impact of each variable in the Cox regression model (**Figure 2**).

### 3.4 Performance of the Radiomics Nomogram

**Figure 3:**
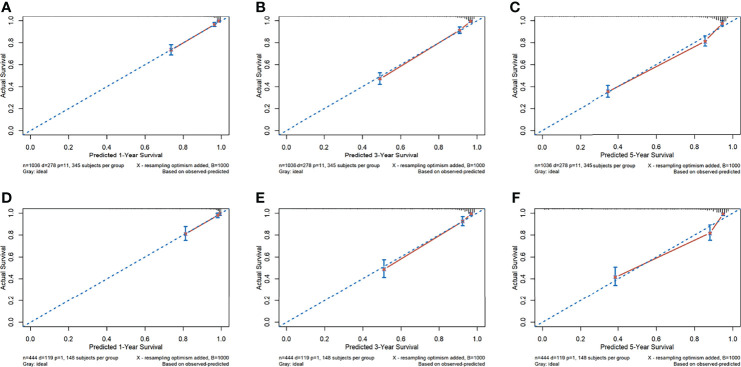
The calibration curves of the radiomics nomogram. (A–F) Calibration curves for predicting patient survival in the training cohort at 1 year **(A)**, 3 years **(B)**, and 5 years **(C)** and in the validation cohort at 1 year **(D)**, 3 years **(E)**, and 5 years **(F)**. The overall survival predicted by the radiomics nomogram is on the *x*-axis, while the actual overall survival is on the *y*-axis. A graph drawn along the diagonal line represents the perfect prediction in which the predicted probabilities is exactly the same as the actual outcomes.

The calibration curves for the probability of 1-, 3-, and 5-year OS showed good agreement between the prediction by the radiomics nomogram and the actual observations in the training cohort and in the validation cohort since the predicted survival probability was very close to the actual survival time of patients (**Figure 3**). Furthermore, based on the radiomics nomogram, we subdivided the patients in the training cohort into four subgroups according to quartiles of predicted survival probabilities. The same threshold was applied to the validation cohort. A significant distinction between Kaplan–Meier curves was found (*p* < 0.001 and *p* < 0.001 in the training cohort and validation cohort, respectively) (**Supplementary Figure S8).**

### 3.5 Assessment of the Incremental Value of Radiomics Signature

The C-index and 95% CI for predicting OS using the clinical model were 0.851 (95% CI: 0.832–0.870) and 0.854 (95% CI: 0.824–0.884) in the training cohort and validation cohort, respectively. The C-index from the radiomics nomogram yielded 0.861 (95% CI: 0.843–0.879) in the training cohort and 0.868 (95% CI: 0.841–0.896) in the validation cohort (**Table 2**). The radiomics nomogram that integrated the radiomics signature and clinical variables outperformed the clinical model based on clinical variables alone, with a *p*-value < 0.001 in the training cohort and *p* = 0.002 in the validation cohort.

## 4 Discussion

Lung cancer is the world’s leading cause of cancer death. Screening for lung cancer by low-dose computed tomography reduces mortality. The NSCLC TNM staging system was developed by the International Association for the Staging of Lung Cancer (IASLC) Lung Cancer Staging Project by a coordinated international effort to develop data-derived TNM classifications with significant survival differences. Based on these TNM groupings, current 5-year survival estimates in NSLCC range from 73% in stage IA disease to 13% in stage IV disease. TNM stage remains the most important prognostic factor in predicting recurrence rates and survival times, followed by tumor histologic grade, and patient sex, age, and performance status (34). However, the wide spectrum of survival times that exists even after complete resection of the same-staged NSCLCs demonstrates the importance of other prognostic factors.

We have developed a radiomics nomogram for predicting the OS of patients with NSCLC in an Eastern Chinese population. The prediction model included the radiomics signatures derived from the CT images using the LASSO Cox regression, and six traditional clinical factors, namely, age, sex, COPD, T stage, N stage, and M stage. The model was developed in the training cohort (*N* = 1,036) and validated in the test dataset (*N* = 444). The model showed good discrimination, which was indicated by the C-index over 0.86 in both the training cohort and the validation cohort. The calibration curves for probability of OS demonstrated good agreement between the prediction by the radiomics nomogram and the actual observations. Risk group stratification further guaranteed the prediction power of the established model, and also confirmed the reliability of our results.

Radiological medical images provide patient and tumor-specific information that could provide insights into personalized medicine and be used to improve clinical prognosis assessment. So far, many studies have demonstrated the effectiveness of radiomics for the prognosis of NSCLC (15, 20, 35). Yang et al. developed a radiomics nomogram by combining the radiomics signatures and four clinical predictors (age, sex, T stage, and N stage) to evaluate the OS with NSCLC patients. The performance of the model was 0.710 measured by the C-index (20). This model was similar to our model, except that we included the M stage as the prognosis factor in the radiomics nomogram (HR = 1.670, 95% CI: 1.254–2.223, *p* < 0.001), and could be applied to a wider range of NSCLC. Moreover, researchers have found that COPD could be a driving factor in lung cancer by increasing oxidative stress and causing DNA damage, inhibiting DNA repair mechanisms, and increasing cell proliferation (36, 37). We have included COPD in our model to make a more precise prediction of OS in NSCLC.

The advantages of our model compared with the other models in predicting NSCLC are listed as follows (1): more comprehensive quantitative features (*N* = 1,288) were extracted from CT images than the previous study, which led to deeper mining of medical imaging; (2) these studies usually employed methods such as the Kaplan–Meier method to clarify the correlation between radiomics features and prognosis. In this study, we used the tenfold cross-validation LASSO Cox model to select the optimal features from 1,288 radiomics features, which contains first-order statistical features, shape-based features, statistical-based texture features, and Gaussian and wavelet information that could improve the stability of the radiomics model. Eleven features were used to construct the radiomics signatures. Our team had successfully established the radiomics nomogram as a preoperative prediction tool for malignant pulmonary nodule diagnosis. The validation results showed that the nomogram has good discrimination (C-index = 0.809) and calibration capacities, which indicated its clinical application in the early screening of lung cancer (24). (3) The sample size (*N* = 1,480) was larger than the previous study; (4) the clinical information was thorough compared with the previous study. We included the sociodemographics of the patients (age at diagnosis, sex, smoking status, and drinking status), the presence of any comorbidity (COPD, hypertension, diabetes, CHD, and cerebrovascular disease), and pathologic data (tumor location, T stage, N stage and M stage) to obtain the optimal prediction clinical variables. Six independent prognostic factors (age, sex, COPD, T stage, N stage, and M stage) were identified by the multivariate Cox model based on the AIC criteria for the best combination to predict the OS and entered into the nomogram; (5) the radiomics signature was an independent prognostic factor and outperformed clinical features in predicting OS of NSCLC patients. Our model yielded a higher C-index of 0.868 (95% CI: 0.841–0.896) in the validation cohort. The performance of our model was measured in a number of ways including calibration and the risk stratification analysis.

In this retrospective study, the data were collected from the Weihai Municipal Hospital located in East China. The characteristics of this Eastern Chinese lung cancer population differ considerably from other, particularly Western, lung cancer populations. On average, people from East China are richer than those in Central and West China, and thanks to widespread screening programs, residents are more willing to undergo routine physical examinations, which is helpful for the early diagnosis of lung cancer. There is a substantial portion of our patients (~60%) who belong to stage I, which inevitably decreases the death rate for the case mix. As shown in **Table 1**, approximately one-third of the patients had a history of smoking and COPD. Female smokers comprise less than 5% of the female patients with lung cancer in our study, which is consistent with a previous study in China and also significantly different from Western lung cancer populations (38). COPD incidence is highly correlated with smoking and female never-smokers comprise a large proportion leading to a lower COPD rate.

In our study, though the radiomics nomogram that integrated the radiomics signature and clinical variables outperformed the clinical model alone (C-index: 0.868 vs. 0.854, *p*-value = 0.002 in validation cohort), the improvement of OS prediction was not very large. Considering that, to some extent, the important prognostic factors (TNM staging, age, etc.) have already been defined. On the other hand, the prediction accuracy of the radiomics signature alone is just slightly lower than the clinical model (C-index: 0.820 vs. 0.854). Therefore, the radiomics signature could also serve as an alternative or auxiliary methodology for the clinical model and confer benefits for the oncologist’s decisions.

Our study also has limitations. First, although there are some studies that apply radiomics to PET-CT image data in lung cancer prognosis prediction, we restricted our radiomics study to CT scans, since currently the CT scans were the primary means for monitoring lung cancer in a real-world clinical environment. Other examination methods are required for comprehensive disease assessment. In the future, we could further target other types of images to evaluate the general condition (such as MRI and ultrasound radiography) and obtain a more precise performance of our model. Secondly, we did not include the treatment variables (such as surgery, radiotherapy, chemotherapy, or targeted therapy) in our study. We hope to include these variables to improve the model performance in a future study. Our model performs well in the absence of treatment information (C-index over 0.86), which also proves the importance of radiomics. Third, the follow-up time was not long enough to obtain each patient’s end point, indicating the heterogeneity of tumor development. Further efforts on patient follow-up are encouraged to improve our model. Finally, this study collected data from a single center; although we divided the validation set to evaluate the stability of model, it is obvious that data from multicenter cohorts and different populations are better. Therefore, further multicenter studies are encouraged to promote the model generalization and improvement. Future prognostication of outcomes in NSCLC will likely be based on a combination of general condition, radiomics, TNM stage, treatment, and molecular tumor profiling, yielding more precise, individualized survival estimates and treatment algorithms.

In summary, we have developed a radiomics nomogram that combines the optimal radiomics signature from CT images with the TNM staging and other clinical information (age, sex and COPD), showing a significant improvement in predicting OS compared with clinical predictors alone. This nomogram should be validated in other Eastern Chinese populations with NSCLC and in other localities, as this work indicates that the radiomics signature increases the precision of survival prediction.

## Data Availability Statement

The original contributions presented in the study are included in the article/**Supplementary Material.** Further inquiries can be directed to the corresponding authors.

## Ethics Statement

The study protocol was conducted under approval by the Public Health Ethics Committee of Shandong University (Approval No. 20180801), The requirement for informed consent was waived because of the retrospective nature of the study. Written informed consent for participation was not required for this study in accordance with the national legislation and the institutional requirements.

## Author Contributions

LW and AL drafted this manuscript. AL, ZW, NX, DZ, GL, and XG collected the data. LW, ZW, and TQ analyzed the data. WC and FX conceived the study and participated in its design and coordination. JW and FY contributed to the interpretation. All authors contributed to the article and approved the submitted version.

## Funding

This work was supported by Key R & D project of Shandong Province (2018GSF118152), the National Key Research and Development Program of China (2020YFC2003500), the National Natural Science Foundation of China (81773547), and the Natural Science Foundation of Shandong Province (ZR2019ZD02).

## Conflict of Interest

The authors declare that the research was conducted in the absence of any commercial or financial relationships that could be construed as a potential conflict of interest.

## Publisher’s Note

All claims expressed in this article are solely those of the authors and do not necessarily represent those of their affiliated organizations, or those of the publisher, the editors and the reviewers. Any product that may be evaluated in this article, or claim that may be made by its manufacturer, is not guaranteed or endorsed by the publisher.

## Acknowledgments

We would like to thank all authors, reviewers, and editors for their critical discussion of this manuscript, and apologize to those not mentioned due to space limitations.

## Abbreviations

NSCLC, non-small cell lung cancer; TNM, tumor-node-metastasis; CT, computed tomography; LASSO, least absolute shrinkage and selection operator; C-index, the Harrell concordance index; HR, hazard ratio; CI, confidence interval.
