# Supplementary material for: A Prognostic Model of Non-Small Cell Lung Cancer With a Radiomics Nomogram in an Eastern Chinese Population
Source: Front Oncol. 2022 Jun 14;12:816766. doi: 10.3389/fonc.2022.816766 (PMC9237399; doi:10.3389/fonc.2022.816766)
Supplement: Supplementary file 1 [file DataSheet_1.pdf]

Supplementary Material

1 Supplementary Material

1.1 Tumor segmentation guidance

Delineation based on CT images was performed by two doctors in Weihai Municipal Hospital, Weihai, Shandong, China (Dr. Ailing Liu, Department of Respiratory Internal Medicine; Dr. Guiyuan Liu, Department of Radiology) who were blinded to the patient cohort using a standard clinical delineation protocol. In our study, we follow the following principles of tumor segmentation:

1) For patients with only one lesion (diameter >= 1cm), the lesions with relatively clear edges were directly selected for segmentation;

2) For patients with multiple lesions on one side of the lung, the lesion with the largest diameter is selected for segmentation;

3) For patients with peripheral lung cancer located in the lung field and away from the chest wall were selected for segmentation;

4) For patients with non-mass lesions were excluded.

Semi-automated segmentation

In the segmentation module, it first used a magic wand-like tool (Level Tracing) in 3D Slicer, put the mouse in the place that the expert thinks is appropriate, moving the mouse defines an outline where the pixels all have the same background value as the current background pixel, the tool will automatically select the appropriate area. If the segmentation wasn't right, the experts could correct it manually using the brush tool and eraser to make certain adjustments according to the situation until the expert thinks it is appropriate. (https://www.slicer.org/wiki/Documentation/4.8/Modules/Editor#Wand).

1.2 Radiomics Features

Due to the excessive number of radiomics features, please refer to the following website for calculation methods for each feature: https://pyradiomics.readthedocs.io/en/latest/features.html.

All radiomics features used in this article were divided into the following three groups:

Group1. First Order Features (18 features, except Standard Deviation)

Group2. Shape Features(3D) (14 features, except Compactness 1 and Compactness 2)

Group3. Texture Features

1) Gray Level Co-occurrence Matrix (GLCM) Features (22 Features, except DEPRECATED. Homogeneity 1and DEPRECATED. Homogeneity 2)

2) Gray Level Size Zone Matrix (GLSZM) Features (16 Features)

3) Gray Level Run Length Matrix (GLRLM) Features (16 Features)

4) Neighbouring Gray Tone Difference Matrix (NGTDM) Features (5 Features)

5) Gray Level Dependence Matrix (GLDM) Features (14 features)

1.3 Image Filters

1.3.1 Laplacian of Gaussian Filter

Applies a Laplacian of Gaussian filter to the input image and yields a derived image for each sigma value specified. A Laplacian of Gaussian image is obtained by convolving the image with the second derivative (Laplacian) of a Gaussian kernel.

The Gaussian kernel is used to smooth the image and is defined as

$$G(x, y, z, \sigma) = \frac{1}{(\sigma\sqrt{2\pi})^3} e^{-\frac{x^2+y^2+z^2}{2\sigma^2}}$$

The Gaussian kernel is convolved by the Laplacian kernel $\nabla^2 G(x, y, z)$, which is sensitive to areas with rapidly changing intensities, enhancing edges. The width of the filter in the Gaussian kernel is determined by σ and can be used to emphasize more fine (low σ values) or coarse (high σ values) textures.

The σ value used in this article was 1,2,3,4,5. We calculated the first order statistics described in Group 1 and the texture features described in Group 3 in an image with this filter.

1.3.2 Wavelet Filter

Wavelet transform effectively decouples texture information by decomposing the original image at low and high frequencies. In this study, a three-dimensional wavelet transform was applied to each CT image, and the original image X was divided into eight decompositions. Think of L and H as low-pass and high-pass functions, respectively, and mark the wavelet decomposition of X as X_{LLL}, X_{LLH}, X_{LHL}, X_{HLL}, X_{LHH}, X_{HHL}, X_{HLH}, X_{HHH}. For example, X_{LLL} as a high-pass sub band, by a low-pass along the x direction Directional filtering of the filter, a low pass filter along the y direction and a low pass filter along the z direction, and constructed as:

$$X_{LLL}(i, j, k) = \sum_{p=1}^{N_L} \sum_{q=1}^{N_L} \sum_{r=1}^{N_L} L(p)L(q)L(r)X(i + p, j + q, k + r)$$

Where N_L is the length of the L filter. Other decompositions are constructed in a similar manner, applying their respective low pass or high pass filtering order in the x, y and z directions. Since the applied wavelet decomposition is unextracted, the size of each decomposition is equal to the original image, and for each decomposition, we calculated the first order statistics described in Group 1 and the texture features described in Group 3.

Therefore, from one original image, five images of Laplacian of Gaussian Filter, and eight wavelet decomposition images for each patient, a total of 1274 radiomics features can be calculated from group 1 and group 3, in addition, 14 features of the Group 2 (shape features) of original images was included, a total of 1288 radiomics features were used in this article.

1.4 The note of selected radiomics features

1) original_shape_Maximum2DDiameterRow

The shape feature named Maximum2DDiameterRow from original image. Maximum 2D diameter (Row) is defined as the largest pairwise Euclidean distance between tumor surface mesh vertices in the column-slice (usually the sagittal) plane.

2) original_shape_Maximum2DDiameterSlice

The shape feature named Maximum2DDiameterSlice from original image. Maximum 2D diameter (Slice) is defined as the largest pairwise Euclidean distance between tumor surface mesh vertices in the row-column (generally the axial) plane.

3) original_firstorder_Median

The first order feature named Median from original image. The median gray level intensity within the ROI.

4) log.sigma.1.0.mm.3D_gldm_DependenceVariance

The GLDM feature named DependenceVariance from image of the Laplacian of Gaussian filter which parameter σ was 1. Dependence Variance (DV)

$$DV = \sum_{i=1}^{N_g} \sum_{j=1}^{N_d} p(i,j)(j-\mu)^2, \text{ where } \mu = \sum_{i=1}^{N_g} \sum_{j=1}^{N_d} jp(i,j)$$

Measures the variance in dependence size in the image.

5) wavelet.LLL_firstorder_Median

The first order feature named Median from image of the Wavelet Filter that a low pass filter along x direction, a low pass filter along the y direction and a low pass filter along the z direction. The median gray level intensity within the ROI.

6) wavelet.LLL_firstorder_RootMeanSquared

The first order feature named RootMeanSquared from image of the Wavelet Filter that a low pass filter along x direction, a low pass filter along the y direction and a low pass filter along the z direction. RMS is the square-root of the mean of all the squared intensity values. It is another measure of the magnitude of the image values.

7) wavelet.LLL_firstorder_Skewness

The first order feature named Skewness from image of the Wavelet Filter that a low pass filter along x direction, a low pass filter along the y direction and a low pass filter along the z direction.

$$\text{skewness} = \frac{\mu_3}{\sigma^3} = \frac{\frac{1}{N_p}\sum_{i=1}^{N_p} (\mathrm{X}(i) - \bar{X})^3}{\left(\sqrt{\frac{1}{N_p}\sum_{i=1}^{N_p} (\mathrm{X}(i) - \bar{X})^2}\right)^3}$$

Where μ_3 is the 3rd central moment.

Skewness measures the asymmetry of the distribution of values about the Mean value.

8) wavelet.LLL_glcm_Idm

The GLCM feature named Idm from image of the Wavelet Filter that a low pass filter along x direction, a low pass filter along the y direction and a low pass filter along the z direction.

$$\text{IDM} = \sum_{k=0}^{N_g-1} \frac{p_{x-y}(k)}{1+k^2}$$

IDM (Inverse Difference Moment) is a measure of the local homogeneity of an image.

9) wavelet.LLL_glcm_InverseVariance

The GLCM feature named InverseVariance from image of the Wavelet Filter that a low pass filter along x direction, a low pass filter along the y direction and a low pass filter along the z direction.

$$\text{inverse variance} = \sum_{k=1}^{N_g-1} \frac{p_{x-y}(k)}{k^2}$$

Note that $k = 0$ is skipped, as this would result in a division by 0.

10) wavelet.LLL_glrlm_RunLengthNonUniformityNormalized

The GLRLM feature named RunLengthNonUniformityNormalized (RLNN) from image of the Wavelet Filter that a low pass filter along x direction, a low pass filter along the y direction and a low pass filter along the z direction.

$$\text{RLNN} = \frac{\sum_{j=1}^{N_r} \left(\sum_{i=1}^{N_g} P(i,j|\theta) \right)^2}{N_r(\theta)^2}$$

RLNN measures the similarity of run lengths throughout the image, with a lower value indicating more homogeneity among run lengths in the image. This is the normalized version of the RLN formula.

11) wavelet.LLL_gldm_DependenceVariance

The GLDM feature named DependenceVariance from image of the Wavelet Filter that a low pass filter along x direction, a low pass filter along the y direction and a low pass filter along the z direction.

$$DV = \sum_{i=1}^{N_g} \sum_{j=1}^{N_d} p(i,j)(j-\mu)^2 \text{, where } \mu = \sum_{i=1}^{N_g} \sum_{j=1}^{N_d} jp(i,j)$$

Measures the variance in dependence size in the image.

2 Supplementary Tables

Supplementary Tables 1. The description of 1288 features.

Features	Image	Groups
original_shape_Elongation	Original	Shape Features
original_shape_Flatness	Original	Shape Features
original_shape_LeastAxisLength	Original	Shape Features
original_shape_MajorAxisLength	Original	Shape Features
original_shape_Maximum2DDiameterColumn	Original	Shape Features
original_shape_Maximum2DDiameterRow	Original	Shape Features
original_shape_Maximum2DDiameterSlice	Original	Shape Features
original_shape_Maximum3DDiameter	Original	Shape Features
original_shape_MeshVolume	Original	Shape Features
original_shape_MinorAxisLength	Original	Shape Features
original_shape_Sphericity	Original	Shape Features
original_shape_SurfaceArea	Original	Shape Features
original_shape_SurfaceVolumeRatio	Original	Shape Features
original_shape_VoxelVolume	Original	Shape Features
original_firstorder_10Percentile	Original	First Order Features
original_firstorder_90Percentile	Original	First Order Features
original_firstorder_Energy	Original	First Order Features

original_firstorder_Entropy	Original	First Order Features
original_firstorder_InterquartileRange	Original	First Order Features
original_firstorder_Kurtosis	Original	First Order Features
original_firstorder_Maximum	Original	First Order Features
original_firstorder_MeanAbsoluteDeviation	Original	First Order Features
original_firstorder_Mean	Original	First Order Features
original_firstorder_Median	Original	First Order Features
original_firstorder_Minimum	Original	First Order Features
original_firstorder_Range	Original	First Order Features
original_firstorder_RobustMeanAbsoluteDeviation	Original	First Order Features
original_firstorder_RootMeanSquared	Original	First Order Features
original_firstorder_Skewness	Original	First Order Features
original_firstorder_TotalEnergy	Original	First Order Features
original_firstorder_Uniformity	Original	First Order Features
original_firstorder_Variance	Original	First Order Features

original_glcm_Autocorrelation	Original	Texture Features--GLCM
original_glcm_JointAverage	Original	Texture Features--GLCM
original_glcm_ClusterProminence	Original	Texture Features--GLCM
original_glcm_ClusterShade	Original	Texture Features--GLCM
original_glcm_ClusterTendency	Original	Texture Features--GLCM
original_glcm_Contrast	Original	Texture Features--GLCM
original_glcm_Correlation	Original	Texture Features--GLCM
original_glcm_DifferenceAverage	Original	Texture Features--GLCM
original_glcm_DifferenceEntropy	Original	Texture Features--GLCM
original_glcm_DifferenceVariance	Original	Texture Features--GLCM
original_glcm_JointEnergy	Original	Texture Features--GLCM
original_glcm_JointEntropy	Original	Texture Features--GLCM
original_glcm_Imc1	Original	Texture Features--GLCM
original_glcm_Imc2	Original	Texture Features--GLCM
original_glcm_Idm	Original	Texture Features--GLCM

original_glcm_Idmn	Original	Texture Features--GLCM
original_glcm_Id	Original	Texture Features--GLCM
original_glcm_Idn	Original	Texture Features--GLCM
original_glcm_InverseVariance	Original	Texture Features--GLCM
original_glcm_MaximumProbability	Original	Texture Features--GLCM
original_glcm_SumEntropy	Original	Texture Features--GLCM
original_glcm_SumSquares	Original	Texture Features--GLCM
original_glrlm_GrayLevelNonUniformity	Original	Texture Features--GLRLM
original_glrlm_GrayLevelNonUniformityNormalized	Original	Texture Features--GLRLM
original_glrlm_GrayLevelVariance	Original	Texture Features--GLRLM
original_glrlm_HighGrayLevelRunEmphasis	Original	Texture Features--GLRLM
original_glrlm_LongRunEmphasis	Original	Texture Features--GLRLM
original_glrlm_LongRunHighGrayLevelEmphasis	Original	Texture Features--GLRLM

original_glrlm_LongRunLowGrayLevelEmphasis	Original	Texture Features--GLRLM
original_glrlm_LowGrayLevelRunEmphasis	Original	Texture Features--GLRLM
original_glrlm_RunEntropy	Original	Texture Features--GLRLM
original_glrlm_RunLengthNonUniformity	Original	Texture Features--GLRLM
original_glrlm_RunLengthNonUniformityNormalized	Original	Texture Features--GLRLM
original_glrlm_RunPercentage	Original	Texture Features--GLRLM
original_glrlm_RunVariance	Original	Texture Features--GLRLM
original_glrlm_ShortRunEmphasis	Original	Texture Features--GLRLM
original_glrlm_ShortRunHighGrayLevelEmphasis	Original	Texture Features--GLRLM
original_glrlm_ShortRunLowGrayLevelEmphasis	Original	Texture Features--GLRLM
original_glszm_GrayLevelNonUniformity	Original	Texture Features--GLSZM
original_glszm_GrayLevelNonUniformityNormalized	Original	Texture Features--GLSZM

original_glszm_GrayLevelVariance	Original	Texture Features-- GLSZM
original_glszm_HighGrayLevelZoneEmphasis	Original	Texture Features-- GLSZM
original_glszm_LargeAreaEmphasis	Original	Texture Features-- GLSZM
original_glszm_LargeAreaHighGrayLevelEmphasis	Original	Texture Features-- GLSZM
original_glszm_LargeAreaLowGrayLevelEmphasis	Original	Texture Features-- GLSZM
original_glszm_LowGrayLevelZoneEmphasis	Original	Texture Features-- GLSZM
original_glszm_SizeZoneNonUniformity	Original	Texture Features-- GLSZM
original_glszm_SizeZoneNonUniformityNormalized	Original	Texture Features-- GLSZM
original_glszm_SmallAreaEmphasis	Original	Texture Features-- GLSZM
original_glszm_SmallAreaHighGrayLevelEmphasis	Original	Texture Features-- GLSZM
original_glszm_SmallAreaLowGrayLevelEmphasis	Original	Texture Features-- GLSZM
original_glszm_ZoneEntropy	Original	Texture Features--

		GLSZM
original_glszm_ZonePercentage	Original	Texture Features--GLSZM
original_glszm_ZoneVariance	Original	Texture Features--GLSZM
original_gldm_DependenceEntropy	Original	Texture Features--GLDM
original_gldm_DependenceNonUniformity	Original	Texture Features--GLDM
original_gldm_DependenceNonUniformityNormalized	Original	Texture Features--GLDM
original_gldm_DependenceVariance	Original	Texture Features--GLDM
original_gldm_GrayLevelNonUniformity	Original	Texture Features--GLDM
original_gldm_GrayLevelVariance	Original	Texture Features--GLDM
original_gldm_HighGrayLevelEmphasis	Original	Texture Features--GLDM
original_gldm_LargeDependenceEmphasis	Original	Texture Features--GLDM
original_gldm_LargeDependenceHighGrayLevelEmphasis	Original	Texture Features--GLDM
original_gldm_LargeDependenceLowGrayLevelEmphasis	Original	Texture Features--

		GLDM
original_gldm_LowGrayLevelEmphasis	Original	Texture Features--GLDM
original_gldm_SmallDependenceEmphasis	Original	Texture Features--GLDM
original_gldm_SmallDependenceHighGrayLevelEmphasis	Original	Texture Features--GLDM
original_gldm_SmallDependenceLowGrayLevelEmphasis	Original	Texture Features--GLDM
original_ngtdm_Busyness	Original	Texture Features--NGTDM
original_ngtdm_Coarseness	Original	Texture Features--NGTDM
original_ngtdm_Complexity	Original	Texture Features--NGTDM
original_ngtdm_Contrast	Original	Texture Features--NGTDM
original_ngtdm_Strength	Original	Texture Features--NGTDM
log_sigma_1mm_3D_firstorder_10Percentile	Laplacian	First Order Features
log_sigma_1mm_3D_firstorder_90Percentile	Laplacian	First Order Features
log_sigma_1mm_3D_firstorder_Energy	Laplacian	First Order Features

log_sigma_1mm_3D_firstorder_Entropy	Laplacian	First Order Features
log_sigma_1mm_3D_firstorder_InterquartileRange	Laplacian	First Order Features
log_sigma_1mm_3D_firstorder_Kurtosis	Laplacian	First Order Features
log_sigma_1mm_3D_firstorder_Maximum	Laplacian	First Order Features
log_sigma_1mm_3D_firstorder_MeanAbsoluteDeviation	Laplacian	First Order Features
log_sigma_1mm_3D_firstorder_Mean	Laplacian	First Order Features
log_sigma_1mm_3D_firstorder_Median	Laplacian	First Order Features
log_sigma_1mm_3D_firstorder_Minimum	Laplacian	First Order Features
log_sigma_1mm_3D_firstorder_Range	Laplacian	First Order Features
log_sigma_1mm_3D_firstorder_RobustMeanAbsoluteDeviation	Laplacian	First Order Features
log_sigma_1mm_3D_firstorder_RootMeanSquared	Laplacian	First Order Features
log_sigma_1mm_3D_firstorder_Skewness	Laplacian	First Order Features
log_sigma_1mm_3D_firstorder_TotalEnergy	Laplacian	First Order Features
log_sigma_1mm_3D_firstorder_Uniformity	Laplacian	First Order Features
log_sigma_1mm_3D_firstorder_Variance	Laplacian	First Order Features

log_sigma_1mm_3D_glcm_Autocorrelation	Laplacian	Texture Features--GLCM
log_sigma_1mm_3D_glcm_JointAverage	Laplacian	Texture Features--GLCM
log_sigma_1mm_3D_glcm_ClusterProminence	Laplacian	Texture Features--GLCM
log_sigma_1mm_3D_glcm_ClusterShade	Laplacian	Texture Features--GLCM
log_sigma_1mm_3D_glcm_ClusterTendency	Laplacian	Texture Features--GLCM
log_sigma_1mm_3D_glcm_Contrast	Laplacian	Texture Features--GLCM
log_sigma_1mm_3D_glcm_Correlation	Laplacian	Texture Features--GLCM
log_sigma_1mm_3D_glcm_DifferenceAverage	Laplacian	Texture Features--GLCM
log_sigma_1mm_3D_glcm_DifferenceEntropy	Laplacian	Texture Features--GLCM
log_sigma_1mm_3D_glcm_DifferenceVariance	Laplacian	Texture Features--GLCM
log_sigma_1mm_3D_glcm_JointEnergy	Laplacian	Texture Features--GLCM
log_sigma_1mm_3D_glcm_JointEntropy	Laplacian	Texture Features--GLCM
log_sigma_1mm_3D_glcm_Imc1	Laplacian	Texture Features--GLCM
log_sigma_1mm_3D_glcm_Imc2	Laplacian	Texture Features--GLCM
log_sigma_1mm_3D_glcm_Idm	Laplacian	Texture Features--GLCM

log_sigma_1mm_3D_glcm_Idmn	Laplacian	Texture Features--GLCM
log_sigma_1mm_3D_glcm_Id	Laplacian	Texture Features--GLCM
log_sigma_1mm_3D_glcm_Idn	Laplacian	Texture Features--GLCM
log_sigma_1mm_3D_glcm_InverseVariance	Laplacian	Texture Features--GLCM
log_sigma_1mm_3D_glcm_MaximumProbability	Laplacian	Texture Features--GLCM
log_sigma_1mm_3D_glcm_SumEntropy	Laplacian	Texture Features--GLCM
log_sigma_1mm_3D_glcm_SumSquares	Laplacian	Texture Features--GLCM
log_sigma_1mm_3D_glrlm_GrayLevelNonUniformity	Laplacian	Texture Features--GLRLM
log_sigma_1mm_3D_glrlm_GrayLevelNonUniformityNormalized	Laplacian	Texture Features--GLRLM
log_sigma_1mm_3D_glrlm_GrayLevelVariance	Laplacian	Texture Features--GLRLM
log_sigma_1mm_3D_glrlm_HighGrayLevelRunEmphasis	Laplacian	Texture Features--GLRLM
log_sigma_1mm_3D_glrlm_LongRunEmphasis	Laplacian	Texture Features--GLRLM
log_sigma_1mm_3D_glrlm_LongRunHighGrayLevelEmphasis	Laplacian	Texture Features--GLRLM
log_sigma_1mm_3D_glrlm_LongRunLowGrayLevelEmphasis	Laplacian	Texture Features--

		GLRLM
log_sigma_1mm_3D_glrlm_LowGrayLevelRunEmphasis	Laplacian	Texture Features-- GLRLM
log_sigma_1mm_3D_glrlm_RunEntropy	Laplacian	Texture Features-- GLRLM
log_sigma_1mm_3D_glrlm_RunLengthNonUniformity	Laplacian	Texture Features-- GLRLM
log_sigma_1mm_3D_glrlm_RunLengthNonUniformityNormalized	Laplacian	Texture Features-- GLRLM
log_sigma_1mm_3D_glrlm_RunPercentage	Laplacian	Texture Features-- GLRLM
log_sigma_1mm_3D_glrlm_RunVariance	Laplacian	Texture Features-- GLRLM
log_sigma_1mm_3D_glrlm_ShortRunEmphasis	Laplacian	Texture Features-- GLRLM
log_sigma_1mm_3D_glrlm_ShortRunHighGrayLevelEmphasis	Laplacian	Texture Features-- GLRLM
log_sigma_1mm_3D_glrlm_ShortRunLowGrayLevelEmphasis	Laplacian	Texture Features-- GLRLM
log_sigma_1mm_3D_glszm_GrayLevelNonUniformity	Laplacian	Texture Features-- GLSZM
log_sigma_1mm_3D_glszm_GrayLevelNonUniformityNormalized	Laplacian	Texture Features-- GLSZM

log_sigma_1mm_3D_glszm_GrayLevelVariance	Laplacian	Texture Features--GLSZM
log_sigma_1mm_3D_glszm_HighGrayLevelZoneEmphasis	Laplacian	Texture Features--GLSZM
log_sigma_1mm_3D_glszm_LargeAreaEmphasis	Laplacian	Texture Features--GLSZM
log_sigma_1mm_3D_glszm_LargeAreaHighGrayLevelEmphasis	Laplacian	Texture Features--GLSZM
log_sigma_1mm_3D_glszm_LargeAreaLowGrayLevelEmphasis	Laplacian	Texture Features--GLSZM
log_sigma_1mm_3D_glszm_LowGrayLevelZoneEmphasis	Laplacian	Texture Features--GLSZM
log_sigma_1mm_3D_glszm_SizeZoneNonUniformity	Laplacian	Texture Features--GLSZM
log_sigma_1mm_3D_glszm_SizeZoneNonUniformityNormalized	Laplacian	Texture Features--GLSZM
log_sigma_1mm_3D_glszm_SmallAreaEmphasis	Laplacian	Texture Features--GLSZM
log_sigma_1mm_3D_glszm_SmallAreaHighGrayLevelEmphasis	Laplacian	Texture Features--GLSZM
log_sigma_1mm_3D_glszm_SmallAreaLowGrayLevelEmphasis	Laplacian	Texture Features--GLSZM
log_sigma_1mm_3D_glszm_ZoneEntropy	Laplacian	Texture Features--GLSZM

log_sigma_1mm_3D_glszm_ZonePercentage	Laplacian	Texture Features--GLSZM
log_sigma_1mm_3D_glszm_ZoneVariance	Laplacian	Texture Features--GLSZM
log_sigma_1mm_3D_gldm_DependenceEntropy	Laplacian	Texture Features--GLDM
log_sigma_1mm_3D_gldm_DependenceNonUniformity	Laplacian	Texture Features--GLDM
log_sigma_1mm_3D_gldm_DependenceNonUniformityNormalized	Laplacian	Texture Features--GLDM
log_sigma_1mm_3D_gldm_DependenceVariance	Laplacian	Texture Features--GLDM
log_sigma_1mm_3D_gldm_GrayLevelNonUniformity	Laplacian	Texture Features--GLDM
log_sigma_1mm_3D_gldm_GrayLevelVariance	Laplacian	Texture Features--GLDM
log_sigma_1mm_3D_gldm_HighGrayLevelEmphasis	Laplacian	Texture Features--GLDM
log_sigma_1mm_3D_gldm_LargeDependenceEmphasis	Laplacian	Texture Features--GLDM
log_sigma_1mm_3D_gldm_LargeDependenceHighGrayLevelEmphasis	Laplacian	Texture Features--GLDM
log_sigma_1mm_3D_gldm_LargeDependenceLowGrayLevelEmphasis	Laplacian	Texture Features--

		GLDM
log_sigma_1mm_3D_gldm_LowGrayLevelEmphasis	Laplacian	Texture Features--GLDM
log_sigma_1mm_3D_gldm_SmallDependenceEmphasis	Laplacian	Texture Features--GLDM
log_sigma_1mm_3D_gldm_SmallDependenceHighGrayLevelEmphasis	Laplacian	Texture Features--GLDM
log_sigma_1mm_3D_gldm_SmallDependenceLowGrayLevelEmphasis	Laplacian	Texture Features--GLDM
log_sigma_1mm_3D_ngtdm_Busyness	Laplacian	Texture Features--NGTDM
log_sigma_1mm_3D_ngtdm_Coarseness	Laplacian	Texture Features--NGTDM
log_sigma_1mm_3D_ngtdm_Complexity	Laplacian	Texture Features--NGTDM
log_sigma_1mm_3D_ngtdm_Contrast	Laplacian	Texture Features--NGTDM
log_sigma_1mm_3D_ngtdm_Strength	Laplacian	Texture Features--NGTDM
log_sigma_2mm_3D_firstorder_10Percentile	Laplacian	First Order Features
log_sigma_2mm_3D_firstorder_90Percentile	Laplacian	First Order Features
log_sigma_2mm_3D_firstorder_Energy	Laplacian	First Order Features

log_sigma_2mm_3D_firstorder_Entropy	Laplacian	First Order Features
log_sigma_2mm_3D_firstorder_InterquartileRange	Laplacian	First Order Features
log_sigma_2mm_3D_firstorder_Kurtosis	Laplacian	First Order Features
log_sigma_2mm_3D_firstorder_Maximum	Laplacian	First Order Features
log_sigma_2mm_3D_firstorder_MeanAbsoluteDeviation	Laplacian	First Order Features
log_sigma_2mm_3D_firstorder_Mean	Laplacian	First Order Features
log_sigma_2mm_3D_firstorder_Median	Laplacian	First Order Features
log_sigma_2mm_3D_firstorder_Minimum	Laplacian	First Order Features
log_sigma_2mm_3D_firstorder_Range	Laplacian	First Order Features
log_sigma_2mm_3D_firstorder_RobustMeanAbsoluteDeviation	Laplacian	First Order Features
log_sigma_2mm_3D_firstorder_RootMeanSquared	Laplacian	First Order Features
log_sigma_2mm_3D_firstorder_Skewness	Laplacian	First Order Features
log_sigma_2mm_3D_firstorder_TotalEnergy	Laplacian	First Order Features
log_sigma_2mm_3D_firstorder_Uniformity	Laplacian	First Order Features
log_sigma_2mm_3D_firstorder_Variance	Laplacian	First Order Features

log_sigma_2mm_3D_glcm_Autocorrelation	Laplacian	Texture Features--GLCM
log_sigma_2mm_3D_glcm_JointAverage	Laplacian	Texture Features--GLCM
log_sigma_2mm_3D_glcm_ClusterProminence	Laplacian	Texture Features--GLCM
log_sigma_2mm_3D_glcm_ClusterShade	Laplacian	Texture Features--GLCM
log_sigma_2mm_3D_glcm_ClusterTendency	Laplacian	Texture Features--GLCM
log_sigma_2mm_3D_glcm_Contrast	Laplacian	Texture Features--GLCM
log_sigma_2mm_3D_glcm_Correlation	Laplacian	Texture Features--GLCM
log_sigma_2mm_3D_glcm_DifferenceAverage	Laplacian	Texture Features--GLCM
log_sigma_2mm_3D_glcm_DifferenceEntropy	Laplacian	Texture Features--GLCM
log_sigma_2mm_3D_glcm_DifferenceVariance	Laplacian	Texture Features--GLCM
log_sigma_2mm_3D_glcm_JointEnergy	Laplacian	Texture Features--GLCM
log_sigma_2mm_3D_glcm_JointEntropy	Laplacian	Texture Features--GLCM
log_sigma_2mm_3D_glcm_Imc1	Laplacian	Texture Features--GLCM
log_sigma_2mm_3D_glcm_Imc2	Laplacian	Texture Features--GLCM
log_sigma_2mm_3D_glcm_Idm	Laplacian	Texture Features--GLCM

log_sigma_2mm_3D_glcm_Idmn	Laplacian	Texture Features--GLCM
log_sigma_2mm_3D_glcm_Id	Laplacian	Texture Features--GLCM
log_sigma_2mm_3D_glcm_Idn	Laplacian	Texture Features--GLCM
log_sigma_2mm_3D_glcm_InverseVariance	Laplacian	Texture Features--GLCM
log_sigma_2mm_3D_glcm_MaximumProbability	Laplacian	Texture Features--GLCM
log_sigma_2mm_3D_glcm_SumEntropy	Laplacian	Texture Features--GLCM
log_sigma_2mm_3D_glcm_SumSquares	Laplacian	Texture Features--GLCM
log_sigma_2mm_3D_glrlm_GrayLevelNonUniformity	Laplacian	Texture Features--GLRLM
log_sigma_2mm_3D_glrlm_GrayLevelNonUniformityNormalized	Laplacian	Texture Features--GLRLM
log_sigma_2mm_3D_glrlm_GrayLevelVariance	Laplacian	Texture Features--GLRLM
log_sigma_2mm_3D_glrlm_HighGrayLevelRunEmphasis	Laplacian	Texture Features--GLRLM
log_sigma_2mm_3D_glrlm_LongRunEmphasis	Laplacian	Texture Features--GLRLM
log_sigma_2mm_3D_glrlm_LongRunHighGrayLevelEmphasis	Laplacian	Texture Features--GLRLM

log_sigma_2mm_3D_glrlm_LongRunLowGrayLevelEmphasis	Laplacian	Texture Features--GLRLM
log_sigma_2mm_3D_glrlm_LowGrayLevelRunEmphasis	Laplacian	Texture Features--GLRLM
log_sigma_2mm_3D_glrlm_RunEntropy	Laplacian	Texture Features--GLRLM
log_sigma_2mm_3D_glrlm_RunLengthNonUniformity	Laplacian	Texture Features--GLRLM
log_sigma_2mm_3D_glrlm_RunLengthNonUniformityNormalized	Laplacian	Texture Features--GLRLM
log_sigma_2mm_3D_glrlm_RunPercentage	Laplacian	Texture Features--GLRLM
log_sigma_2mm_3D_glrlm_RunVariance	Laplacian	Texture Features--GLRLM
log_sigma_2mm_3D_glrlm_ShortRunEmphasis	Laplacian	Texture Features--GLRLM
log_sigma_2mm_3D_glrlm_ShortRunHighGrayLevelEmphasis	Laplacian	Texture Features--GLRLM
log_sigma_2mm_3D_glrlm_ShortRunLowGrayLevelEmphasis	Laplacian	Texture Features--GLRLM
log_sigma_2mm_3D_glszm_GrayLevelNonUniformity	Laplacian	Texture Features--GLSZM
log_sigma_2mm_3D_glszm_GrayLevelNonUniformityNormalized	Laplacian	Texture Features--GLSZM

log_sigma_2mm_3D_glszm_GrayLevelVariance	Laplacian	Texture Features--GLSZM
log_sigma_2mm_3D_glszm_HighGrayLevelZoneEmphasis	Laplacian	Texture Features--GLSZM
log_sigma_2mm_3D_glszm_LargeAreaEmphasis	Laplacian	Texture Features--GLSZM
log_sigma_2mm_3D_glszm_LargeAreaHighGrayLevelEmphasis	Laplacian	Texture Features--GLSZM
log_sigma_2mm_3D_glszm_LargeAreaLowGrayLevelEmphasis	Laplacian	Texture Features--GLSZM
log_sigma_2mm_3D_glszm_LowGrayLevelZoneEmphasis	Laplacian	Texture Features--GLSZM
log_sigma_2mm_3D_glszm_SizeZoneNonUniformity	Laplacian	Texture Features--GLSZM
log_sigma_2mm_3D_glszm_SizeZoneNonUniformityNormalized	Laplacian	Texture Features--GLSZM
log_sigma_2mm_3D_glszm_SmallAreaEmphasis	Laplacian	Texture Features--GLSZM
log_sigma_2mm_3D_glszm_SmallAreaHighGrayLevelEmphasis	Laplacian	Texture Features--GLSZM
log_sigma_2mm_3D_glszm_SmallAreaLowGrayLevelEmphasis	Laplacian	Texture Features--GLSZM
log_sigma_2mm_3D_glszm_ZoneEntropy	Laplacian	Texture Features--

		GLSZM
log_sigma_2mm_3D_glszm_ZonePercentage	Laplacian	Texture Features-- GLSZM
log_sigma_2mm_3D_glszm_ZoneVariance	Laplacian	Texture Features-- GLSZM
log_sigma_2mm_3D_gldm_DependenceEntropy	Laplacian	Texture Features-- GLDM
log_sigma_2mm_3D_gldm_DependenceNonUniformity	Laplacian	Texture Features-- GLDM
log_sigma_2mm_3D_gldm_DependenceNonUniformityNormalized	Laplacian	Texture Features-- GLDM
log_sigma_2mm_3D_gldm_DependenceVariance	Laplacian	Texture Features-- GLDM
log_sigma_2mm_3D_gldm_GrayLevelNonUniformity	Laplacian	Texture Features-- GLDM
log_sigma_2mm_3D_gldm_GrayLevelVariance	Laplacian	Texture Features-- GLDM
log_sigma_2mm_3D_gldm_HighGrayLevelEmphasis	Laplacian	Texture Features-- GLDM
log_sigma_2mm_3D_gldm_LargeDependenceEmphasis	Laplacian	Texture Features-- GLDM
log_sigma_2mm_3D_gldm_LargeDependenceHighGrayLevelEmphasis	Laplacian	Texture Features-- GLDM
log_sigma_2mm_3D_gldm_LargeDependenceLowGrayLevelEmphasis	Laplacian	Texture Features--

		GLDM
log_sigma_2mm_3D_gldm_LowGrayLevelEmphasis	Laplacian	Texture Features--GLDM
log_sigma_2mm_3D_gldm_SmallDependenceEmphasis	Laplacian	Texture Features--GLDM
log_sigma_2mm_3D_gldm_SmallDependenceHighGrayLevelEmphasis	Laplacian	Texture Features--GLDM
log_sigma_2mm_3D_gldm_SmallDependenceLowGrayLevelEmphasis	Laplacian	Texture Features--GLDM
log_sigma_2mm_3D_ngtdm_Busyness	Laplacian	Texture Features--NGTDM
log_sigma_2mm_3D_ngtdm_Coarseness	Laplacian	Texture Features--NGTDM
log_sigma_2mm_3D_ngtdm_Complexity	Laplacian	Texture Features--NGTDM
log_sigma_2mm_3D_ngtdm_Contrast	Laplacian	Texture Features--NGTDM
log_sigma_2mm_3D_ngtdm_Strength	Laplacian	Texture Features--NGTDM
log_sigma_3mm_3D_firstorder_10Percentile	Laplacian	First Order Features
log_sigma_3mm_3D_firstorder_90Percentile	Laplacian	First Order Features
log_sigma_3mm_3D_firstorder_Energy	Laplacian	First Order Features

log_sigma_3mm_3D_firstorder_Entropy	Laplacian	First Order Features
log_sigma_3mm_3D_firstorder_InterquartileRange	Laplacian	First Order Features
log_sigma_3mm_3D_firstorder_Kurtosis	Laplacian	First Order Features
log_sigma_3mm_3D_firstorder_Maximum	Laplacian	First Order Features
log_sigma_3mm_3D_firstorder_MeanAbsoluteDeviation	Laplacian	First Order Features
log_sigma_3mm_3D_firstorder_Mean	Laplacian	First Order Features
log_sigma_3mm_3D_firstorder_Median	Laplacian	First Order Features
log_sigma_3mm_3D_firstorder_Minimum	Laplacian	First Order Features
log_sigma_3mm_3D_firstorder_Range	Laplacian	First Order Features
log_sigma_3mm_3D_firstorder_RobustMeanAbsoluteDeviation	Laplacian	First Order Features
log_sigma_3mm_3D_firstorder_RootMeanSquared	Laplacian	First Order Features
log_sigma_3mm_3D_firstorder_Skewness	Laplacian	First Order Features
log_sigma_3mm_3D_firstorder_TotalEnergy	Laplacian	First Order Features
log_sigma_3mm_3D_firstorder_Uniformity	Laplacian	First Order Features
log_sigma_3mm_3D_firstorder_Variance	Laplacian	First Order Features

log_sigma_3mm_3D_glcm_Autocorrelation	Laplacian	Texture Features--GLCM
log_sigma_3mm_3D_glcm_JointAverage	Laplacian	Texture Features--GLCM
log_sigma_3mm_3D_glcm_ClusterProminence	Laplacian	Texture Features--GLCM
log_sigma_3mm_3D_glcm_ClusterShade	Laplacian	Texture Features--GLCM
log_sigma_3mm_3D_glcm_ClusterTendency	Laplacian	Texture Features--GLCM
log_sigma_3mm_3D_glcm_Contrast	Laplacian	Texture Features--GLCM
log_sigma_3mm_3D_glcm_Correlation	Laplacian	Texture Features--GLCM
log_sigma_3mm_3D_glcm_DifferenceAverage	Laplacian	Texture Features--GLCM
log_sigma_3mm_3D_glcm_DifferenceEntropy	Laplacian	Texture Features--GLCM
log_sigma_3mm_3D_glcm_DifferenceVariance	Laplacian	Texture Features--GLCM
log_sigma_3mm_3D_glcm_JointEnergy	Laplacian	Texture Features--GLCM
log_sigma_3mm_3D_glcm_JointEntropy	Laplacian	Texture Features--GLCM
log_sigma_3mm_3D_glcm_Imc1	Laplacian	Texture Features--GLCM
log_sigma_3mm_3D_glcm_Imc2	Laplacian	Texture Features--GLCM
log_sigma_3mm_3D_glcm_Idm	Laplacian	Texture Features--GLCM

log_sigma_3mm_3D_glcm_Idmn	Laplacian	Texture Features--GLCM
log_sigma_3mm_3D_glcm_Id	Laplacian	Texture Features--GLCM
log_sigma_3mm_3D_glcm_Idn	Laplacian	Texture Features--GLCM
log_sigma_3mm_3D_glcm_InverseVariance	Laplacian	Texture Features--GLCM
log_sigma_3mm_3D_glcm_MaximumProbability	Laplacian	Texture Features--GLCM
log_sigma_3mm_3D_glcm_SumEntropy	Laplacian	Texture Features--GLCM
log_sigma_3mm_3D_glcm_SumSquares	Laplacian	Texture Features--GLCM
log_sigma_3mm_3D_glrlm_GrayLevelNonUniformity	Laplacian	Texture Features--GLRLM
log_sigma_3mm_3D_glrlm_GrayLevelNonUniformityNormalized	Laplacian	Texture Features--GLRLM
log_sigma_3mm_3D_glrlm_GrayLevelVariance	Laplacian	Texture Features--GLRLM
log_sigma_3mm_3D_glrlm_HighGrayLevelRunEmphasis	Laplacian	Texture Features--GLRLM
log_sigma_3mm_3D_glrlm_LongRunEmphasis	Laplacian	Texture Features--GLRLM
log_sigma_3mm_3D_glrlm_LongRunHighGrayLevelEmphasis	Laplacian	Texture Features--GLRLM
log_sigma_3mm_3D_glrlm_LongRunLowGrayLevelEmphasis	Laplacian	Texture Features--

		GLRLM
log_sigma_3mm_3D_glrlm_LowGrayLevelRunEmphasis	Laplacian	Texture Features-- GLRLM
log_sigma_3mm_3D_glrlm_RunEntropy	Laplacian	Texture Features-- GLRLM
log_sigma_3mm_3D_glrlm_RunLengthNonUniformity	Laplacian	Texture Features-- GLRLM
log_sigma_3mm_3D_glrlm_RunLengthNonUniformityNormalized	Laplacian	Texture Features-- GLRLM
log_sigma_3mm_3D_glrlm_RunPercentage	Laplacian	Texture Features-- GLRLM
log_sigma_3mm_3D_glrlm_RunVariance	Laplacian	Texture Features-- GLRLM
log_sigma_3mm_3D_glrlm_ShortRunEmphasis	Laplacian	Texture Features-- GLRLM
log_sigma_3mm_3D_glrlm_ShortRunHighGrayLevelEmphasis	Laplacian	Texture Features-- GLRLM
log_sigma_3mm_3D_glrlm_ShortRunLowGrayLevelEmphasis	Laplacian	Texture Features-- GLRLM
log_sigma_3mm_3D_glszm_GrayLevelNonUniformity	Laplacian	Texture Features-- GLSZM
log_sigma_3mm_3D_glszm_GrayLevelNonUniformityNormalized	Laplacian	Texture Features-- GLSZM

log_sigma_3mm_3D_glszm_GrayLevelVariance	Laplacian	Texture Features--GLSZM
log_sigma_3mm_3D_glszm_HighGrayLevelZoneEmphasis	Laplacian	Texture Features--GLSZM
log_sigma_3mm_3D_glszm_LargeAreaEmphasis	Laplacian	Texture Features--GLSZM
log_sigma_3mm_3D_glszm_LargeAreaHighGrayLevelEmphasis	Laplacian	Texture Features--GLSZM
log_sigma_3mm_3D_glszm_LargeAreaLowGrayLevelEmphasis	Laplacian	Texture Features--GLSZM
log_sigma_3mm_3D_glszm_LowGrayLevelZoneEmphasis	Laplacian	Texture Features--GLSZM
log_sigma_3mm_3D_glszm_SizeZoneNonUniformity	Laplacian	Texture Features--GLSZM
log_sigma_3mm_3D_glszm_SizeZoneNonUniformityNormalized	Laplacian	Texture Features--GLSZM
log_sigma_3mm_3D_glszm_SmallAreaEmphasis	Laplacian	Texture Features--GLSZM
log_sigma_3mm_3D_glszm_SmallAreaHighGrayLevelEmphasis	Laplacian	Texture Features--GLSZM
log_sigma_3mm_3D_glszm_SmallAreaLowGrayLevelEmphasis	Laplacian	Texture Features--GLSZM
log_sigma_3mm_3D_glszm_ZoneEntropy	Laplacian	Texture Features--GLSZM

log_sigma_3mm_3D_glszm_ZonePercentage	Laplacian	Texture Features-- GLSZM
log_sigma_3mm_3D_glszm_ZoneVariance	Laplacian	Texture Features-- GLSZM
log_sigma_3mm_3D_gldm_DependenceEntropy	Laplacian	Texture Features-- GLDM
log_sigma_3mm_3D_gldm_DependenceNonUniformity	Laplacian	Texture Features-- GLDM
log_sigma_3mm_3D_gldm_DependenceNonUniformityNormalized	Laplacian	Texture Features-- GLDM
log_sigma_3mm_3D_gldm_DependenceVariance	Laplacian	Texture Features-- GLDM
log_sigma_3mm_3D_gldm_GrayLevelNonUniformity	Laplacian	Texture Features-- GLDM
log_sigma_3mm_3D_gldm_GrayLevelVariance	Laplacian	Texture Features-- GLDM
log_sigma_3mm_3D_gldm_HighGrayLevelEmphasis	Laplacian	Texture Features-- GLDM
log_sigma_3mm_3D_gldm_LargeDependenceEmphasis	Laplacian	Texture Features-- GLDM
log_sigma_3mm_3D_gldm_LargeDependenceHighGrayLevelEmphasis	Laplacian	Texture Features-- GLDM
log_sigma_3mm_3D_gldm_LargeDependenceLowGrayLevelEmphasis	Laplacian	Texture Features--

		GLDM
log_sigma_3mm_3D_gldm_LowGrayLevelEmphasis	Laplacian	Texture Features--GLDM
log_sigma_3mm_3D_gldm_SmallDependenceEmphasis	Laplacian	Texture Features--GLDM
log_sigma_3mm_3D_gldm_SmallDependenceHighGrayLevelEmphasis	Laplacian	Texture Features--GLDM
log_sigma_3mm_3D_gldm_SmallDependenceLowGrayLevelEmphasis	Laplacian	Texture Features--GLDM
log_sigma_3mm_3D_ngtdm_Busyness	Laplacian	Texture Features--NGTDM
log_sigma_3mm_3D_ngtdm_Coarseness	Laplacian	Texture Features--NGTDM
log_sigma_3mm_3D_ngtdm_Complexity	Laplacian	Texture Features--NGTDM
log_sigma_3mm_3D_ngtdm_Contrast	Laplacian	Texture Features--NGTDM
log_sigma_3mm_3D_ngtdm_Strength	Laplacian	Texture Features--NGTDM
log_sigma_4mm_3D_firstorder_10Percentile	Laplacian	First Order Features
log_sigma_4mm_3D_firstorder_90Percentile	Laplacian	First Order Features
log_sigma_4mm_3D_firstorder_Energy	Laplacian	First Order Features

log_sigma_4mm_3D_firstorder_Entropy	Laplacian	First Order Features
log_sigma_4mm_3D_firstorder_InterquartileRange	Laplacian	First Order Features
log_sigma_4mm_3D_firstorder_Kurtosis	Laplacian	First Order Features
log_sigma_4mm_3D_firstorder_Maximum	Laplacian	First Order Features
log_sigma_4mm_3D_firstorder_MeanAbsoluteDeviation	Laplacian	First Order Features
log_sigma_4mm_3D_firstorder_Mean	Laplacian	First Order Features
log_sigma_4mm_3D_firstorder_Median	Laplacian	First Order Features
log_sigma_4mm_3D_firstorder_Minimum	Laplacian	First Order Features
log_sigma_4mm_3D_firstorder_Range	Laplacian	First Order Features
log_sigma_4mm_3D_firstorder_RobustMeanAbsoluteDeviation	Laplacian	First Order Features
log_sigma_4mm_3D_firstorder_RootMeanSquared	Laplacian	First Order Features
log_sigma_4mm_3D_firstorder_Skewness	Laplacian	First Order Features
log_sigma_4mm_3D_firstorder_TotalEnergy	Laplacian	First Order Features
log_sigma_4mm_3D_firstorder_Uniformity	Laplacian	First Order Features
log_sigma_4mm_3D_firstorder_Variance	Laplacian	First Order Features

log_sigma_4mm_3D_glcm_Autocorrelation	Laplacian	Texture Features--GLCM
log_sigma_4mm_3D_glcm_JointAverage	Laplacian	Texture Features--GLCM
log_sigma_4mm_3D_glcm_ClusterProminence	Laplacian	Texture Features--GLCM
log_sigma_4mm_3D_glcm_ClusterShade	Laplacian	Texture Features--GLCM
log_sigma_4mm_3D_glcm_ClusterTendency	Laplacian	Texture Features--GLCM
log_sigma_4mm_3D_glcm_Contrast	Laplacian	Texture Features--GLCM
log_sigma_4mm_3D_glcm_Correlation	Laplacian	Texture Features--GLCM
log_sigma_4mm_3D_glcm_DifferenceAverage	Laplacian	Texture Features--GLCM
log_sigma_4mm_3D_glcm_DifferenceEntropy	Laplacian	Texture Features--GLCM
log_sigma_4mm_3D_glcm_DifferenceVariance	Laplacian	Texture Features--GLCM
log_sigma_4mm_3D_glcm_JointEnergy	Laplacian	Texture Features--GLCM
log_sigma_4mm_3D_glcm_JointEntropy	Laplacian	Texture Features--GLCM
log_sigma_4mm_3D_glcm_Imc1	Laplacian	Texture Features--GLCM
log_sigma_4mm_3D_glcm_Imc2	Laplacian	Texture Features--GLCM
log_sigma_4mm_3D_glcm_Idm	Laplacian	Texture Features--GLCM

log_sigma_4mm_3D_glcm_Idmn	Laplacian	Texture Features--GLCM
log_sigma_4mm_3D_glcm_Id	Laplacian	Texture Features--GLCM
log_sigma_4mm_3D_glcm_Idn	Laplacian	Texture Features--GLCM
log_sigma_4mm_3D_glcm_InverseVariance	Laplacian	Texture Features--GLCM
log_sigma_4mm_3D_glcm_MaximumProbability	Laplacian	Texture Features--GLCM
log_sigma_4mm_3D_glcm_SumEntropy	Laplacian	Texture Features--GLCM
log_sigma_4mm_3D_glcm_SumSquares	Laplacian	Texture Features--GLCM
log_sigma_4mm_3D_glrlm_GrayLevelNonUniformity	Laplacian	Texture Features--GLRLM
log_sigma_4mm_3D_glrlm_GrayLevelNonUniformityNormalized	Laplacian	Texture Features--GLRLM
log_sigma_4mm_3D_glrlm_GrayLevelVariance	Laplacian	Texture Features--GLRLM
log_sigma_4mm_3D_glrlm_HighGrayLevelRunEmphasis	Laplacian	Texture Features--GLRLM
log_sigma_4mm_3D_glrlm_LongRunEmphasis	Laplacian	Texture Features--GLRLM
log_sigma_4mm_3D_glrlm_LongRunHighGrayLevelEmphasis	Laplacian	Texture Features--GLRLM

log_sigma_4mm_3D_glrlm_LongRunLowGrayLevelEmphasis	Laplacian	Texture Features--GLRLM
log_sigma_4mm_3D_glrlm_LowGrayLevelRunEmphasis	Laplacian	Texture Features--GLRLM
log_sigma_4mm_3D_glrlm_RunEntropy	Laplacian	Texture Features--GLRLM
log_sigma_4mm_3D_glrlm_RunLengthNonUniformity	Laplacian	Texture Features--GLRLM
log_sigma_4mm_3D_glrlm_RunLengthNonUniformityNormalized	Laplacian	Texture Features--GLRLM
log_sigma_4mm_3D_glrlm_RunPercentage	Laplacian	Texture Features--GLRLM
log_sigma_4mm_3D_glrlm_RunVariance	Laplacian	Texture Features--GLRLM
log_sigma_4mm_3D_glrlm_ShortRunEmphasis	Laplacian	Texture Features--GLRLM
log_sigma_4mm_3D_glrlm_ShortRunHighGrayLevelEmphasis	Laplacian	Texture Features--GLRLM
log_sigma_4mm_3D_glrlm_ShortRunLowGrayLevelEmphasis	Laplacian	Texture Features--GLRLM
log_sigma_4mm_3D_glszm_GrayLevelNonUniformity	Laplacian	Texture Features--GLSZM
log_sigma_4mm_3D_glszm_GrayLevelNonUniformityNormalized	Laplacian	Texture Features--GLSZM

log_sigma_4mm_3D_glszm_GrayLevelVariance	Laplacian	Texture Features--GLSZM
log_sigma_4mm_3D_glszm_HighGrayLevelZoneEmphasis	Laplacian	Texture Features--GLSZM
log_sigma_4mm_3D_glszm_LargeAreaEmphasis	Laplacian	Texture Features--GLSZM
log_sigma_4mm_3D_glszm_LargeAreaHighGrayLevelEmphasis	Laplacian	Texture Features--GLSZM
log_sigma_4mm_3D_glszm_LargeAreaLowGrayLevelEmphasis	Laplacian	Texture Features--GLSZM
log_sigma_4mm_3D_glszm_LowGrayLevelZoneEmphasis	Laplacian	Texture Features--GLSZM
log_sigma_4mm_3D_glszm_SizeZoneNonUniformity	Laplacian	Texture Features--GLSZM
log_sigma_4mm_3D_glszm_SizeZoneNonUniformityNormalized	Laplacian	Texture Features--GLSZM
log_sigma_4mm_3D_glszm_SmallAreaEmphasis	Laplacian	Texture Features--GLSZM
log_sigma_4mm_3D_glszm_SmallAreaHighGrayLevelEmphasis	Laplacian	Texture Features--GLSZM
log_sigma_4mm_3D_glszm_SmallAreaLowGrayLevelEmphasis	Laplacian	Texture Features--GLSZM
log_sigma_4mm_3D_glszm_ZoneEntropy	Laplacian	Texture Features--

		GLSZM
log_sigma_4mm_3D_glszm_ZonePercentage	Laplacian	Texture Features-- GLSZM
log_sigma_4mm_3D_glszm_ZoneVariance	Laplacian	Texture Features-- GLSZM
log_sigma_4mm_3D_gldm_DependenceEntropy	Laplacian	Texture Features-- GLDM
log_sigma_4mm_3D_gldm_DependenceNonUniformity	Laplacian	Texture Features-- GLDM
log_sigma_4mm_3D_gldm_DependenceNonUniformityNormalized	Laplacian	Texture Features-- GLDM
log_sigma_4mm_3D_gldm_DependenceVariance	Laplacian	Texture Features-- GLDM
log_sigma_4mm_3D_gldm_GrayLevelNonUniformity	Laplacian	Texture Features-- GLDM
log_sigma_4mm_3D_gldm_GrayLevelVariance	Laplacian	Texture Features-- GLDM
log_sigma_4mm_3D_gldm_HighGrayLevelEmphasis	Laplacian	Texture Features-- GLDM
log_sigma_4mm_3D_gldm_LargeDependenceEmphasis	Laplacian	Texture Features-- GLDM
log_sigma_4mm_3D_gldm_LargeDependenceHighGrayLevelEmphasis	Laplacian	Texture Features-- GLDM
log_sigma_4mm_3D_gldm_LargeDependenceLowGrayLevelEmphasis	Laplacian	Texture Features--

		GLDM
log_sigma_4mm_3D_gldm_LowGrayLevelEmphasis	Laplacian	Texture Features-- GLDM
log_sigma_4mm_3D_gldm_SmallDependenceEmphasis	Laplacian	Texture Features-- GLDM
log_sigma_4mm_3D_gldm_SmallDependenceHighGrayLevelEmphasis	Laplacian	Texture Features-- GLDM
log_sigma_4mm_3D_gldm_SmallDependenceLowGrayLevelEmphasis	Laplacian	Texture Features-- GLDM
log_sigma_4mm_3D_ngtdm_Busyness	Laplacian	Texture Features-- NGTDM
log_sigma_4mm_3D_ngtdm_Coarseness	Laplacian	Texture Features-- NGTDM
log_sigma_4mm_3D_ngtdm_Complexity	Laplacian	Texture Features-- NGTDM
log_sigma_4mm_3D_ngtdm_Contrast	Laplacian	Texture Features-- NGTDM
log_sigma_4mm_3D_ngtdm_Strength	Laplacian	Texture Features-- NGTDM
log_sigma_5mm_3D_firstorder_10Percentile	Laplacian	First Order Features
log_sigma_5mm_3D_firstorder_90Percentile	Laplacian	First Order Features
log_sigma_5mm_3D_firstorder_Energy	Laplacian	First Order Features

log_sigma_5mm_3D_firstorder_Entropy	Laplacian	First Order Features
log_sigma_5mm_3D_firstorder_InterquartileRange	Laplacian	First Order Features
log_sigma_5mm_3D_firstorder_Kurtosis	Laplacian	First Order Features
log_sigma_5mm_3D_firstorder_Maximum	Laplacian	First Order Features
log_sigma_5mm_3D_firstorder_MeanAbsoluteDeviation	Laplacian	First Order Features
log_sigma_5mm_3D_firstorder_Mean	Laplacian	First Order Features
log_sigma_5mm_3D_firstorder_Median	Laplacian	First Order Features
log_sigma_5mm_3D_firstorder_Minimum	Laplacian	First Order Features
log_sigma_5mm_3D_firstorder_Range	Laplacian	First Order Features
log_sigma_5mm_3D_firstorder_RobustMeanAbsoluteDeviation	Laplacian	First Order Features
log_sigma_5mm_3D_firstorder_RootMeanSquared	Laplacian	First Order Features
log_sigma_5mm_3D_firstorder_Skewness	Laplacian	First Order Features
log_sigma_5mm_3D_firstorder_TotalEnergy	Laplacian	First Order Features
log_sigma_5mm_3D_firstorder_Uniformity	Laplacian	First Order Features
log_sigma_5mm_3D_firstorder_Variance	Laplacian	First Order Features

log_sigma_5mm_3D_glcm_Autocorrelation	Laplacian	Texture Features--GLCM
log_sigma_5mm_3D_glcm_JointAverage	Laplacian	Texture Features--GLCM
log_sigma_5mm_3D_glcm_ClusterProminence	Laplacian	Texture Features--GLCM
log_sigma_5mm_3D_glcm_ClusterShade	Laplacian	Texture Features--GLCM
log_sigma_5mm_3D_glcm_ClusterTendency	Laplacian	Texture Features--GLCM
log_sigma_5mm_3D_glcm_Contrast	Laplacian	Texture Features--GLCM
log_sigma_5mm_3D_glcm_Correlation	Laplacian	Texture Features--GLCM
log_sigma_5mm_3D_glcm_DifferenceAverage	Laplacian	Texture Features--GLCM
log_sigma_5mm_3D_glcm_DifferenceEntropy	Laplacian	Texture Features--GLCM
log_sigma_5mm_3D_glcm_DifferenceVariance	Laplacian	Texture Features--GLCM
log_sigma_5mm_3D_glcm_JointEnergy	Laplacian	Texture Features--GLCM
log_sigma_5mm_3D_glcm_JointEntropy	Laplacian	Texture Features--GLCM
log_sigma_5mm_3D_glcm_Imc1	Laplacian	Texture Features--GLCM
log_sigma_5mm_3D_glcm_Imc2	Laplacian	Texture Features--GLCM
log_sigma_5mm_3D_glcm_Idm	Laplacian	Texture Features--GLCM

log_sigma_5mm_3D_glcm_Idmn	Laplacian	Texture Features--GLCM
log_sigma_5mm_3D_glcm_Id	Laplacian	Texture Features--GLCM
log_sigma_5mm_3D_glcm_Idn	Laplacian	Texture Features--GLCM
log_sigma_5mm_3D_glcm_InverseVariance	Laplacian	Texture Features--GLCM
log_sigma_5mm_3D_glcm_MaximumProbability	Laplacian	Texture Features--GLCM
log_sigma_5mm_3D_glcm_SumEntropy	Laplacian	Texture Features--GLCM
log_sigma_5mm_3D_glcm_SumSquares	Laplacian	Texture Features--GLCM
log_sigma_5mm_3D_glrlm_GrayLevelNonUniformity	Laplacian	Texture Features--GLRLM
log_sigma_5mm_3D_glrlm_GrayLevelNonUniformityNormalized	Laplacian	Texture Features--GLRLM
log_sigma_5mm_3D_glrlm_GrayLevelVariance	Laplacian	Texture Features--GLRLM
log_sigma_5mm_3D_glrlm_HighGrayLevelRunEmphasis	Laplacian	Texture Features--GLRLM
log_sigma_5mm_3D_glrlm_LongRunEmphasis	Laplacian	Texture Features--GLRLM
log_sigma_5mm_3D_glrlm_LongRunHighGrayLevelEmphasis	Laplacian	Texture Features--GLRLM
log_sigma_5mm_3D_glrlm_LongRunLowGrayLevelEmphasis	Laplacian	Texture Features--

		GLRLM
log_sigma_5mm_3D_glrlm_LowGrayLevelRunEmphasis	Laplacian	Texture Features-- GLRLM
log_sigma_5mm_3D_glrlm_RunEntropy	Laplacian	Texture Features-- GLRLM
log_sigma_5mm_3D_glrlm_RunLengthNonUniformity	Laplacian	Texture Features-- GLRLM
log_sigma_5mm_3D_glrlm_RunLengthNonUniformityNormalized	Laplacian	Texture Features-- GLRLM
log_sigma_5mm_3D_glrlm_RunPercentage	Laplacian	Texture Features-- GLRLM
log_sigma_5mm_3D_glrlm_RunVariance	Laplacian	Texture Features-- GLRLM
log_sigma_5mm_3D_glrlm_ShortRunEmphasis	Laplacian	Texture Features-- GLRLM
log_sigma_5mm_3D_glrlm_ShortRunHighGrayLevelEmphasis	Laplacian	Texture Features-- GLRLM
log_sigma_5mm_3D_glrlm_ShortRunLowGrayLevelEmphasis	Laplacian	Texture Features-- GLRLM
log_sigma_5mm_3D_glszm_GrayLevelNonUniformity	Laplacian	Texture Features-- GLSZM
log_sigma_5mm_3D_glszm_GrayLevelNonUniformityNormalized	Laplacian	Texture Features-- GLSZM

log_sigma_5mm_3D_glszm_GrayLevelVariance	Laplacian	Texture Features-- GLSZM
log_sigma_5mm_3D_glszm_HighGrayLevelZoneEmphasis	Laplacian	Texture Features-- GLSZM
log_sigma_5mm_3D_glszm_LargeAreaEmphasis	Laplacian	Texture Features-- GLSZM
log_sigma_5mm_3D_glszm_LargeAreaHighGrayLevelEmphasis	Laplacian	Texture Features-- GLSZM
log_sigma_5mm_3D_glszm_LargeAreaLowGrayLevelEmphasis	Laplacian	Texture Features-- GLSZM
log_sigma_5mm_3D_glszm_LowGrayLevelZoneEmphasis	Laplacian	Texture Features-- GLSZM
log_sigma_5mm_3D_glszm_SizeZoneNonUniformity	Laplacian	Texture Features-- GLSZM
log_sigma_5mm_3D_glszm_SizeZoneNonUniformityNormalized	Laplacian	Texture Features-- GLSZM
log_sigma_5mm_3D_glszm_SmallAreaEmphasis	Laplacian	Texture Features-- GLSZM
log_sigma_5mm_3D_glszm_SmallAreaHighGrayLevelEmphasis	Laplacian	Texture Features-- GLSZM
log_sigma_5mm_3D_glszm_SmallAreaLowGrayLevelEmphasis	Laplacian	Texture Features-- GLSZM
log_sigma_5mm_3D_glszm_ZoneEntropy	Laplacian	Texture Features-- GLSZM

log_sigma_5mm_3D_glszm_ZonePercentage	Laplacian	Texture Features--GLSZM
log_sigma_5mm_3D_glszm_ZoneVariance	Laplacian	Texture Features--GLSZM
log_sigma_5mm_3D_gldm_DependenceEntropy	Laplacian	Texture Features--GLDM
log_sigma_5mm_3D_gldm_DependenceNonUniformity	Laplacian	Texture Features--GLDM
log_sigma_5mm_3D_gldm_DependenceNonUniformityNormalized	Laplacian	Texture Features--GLDM
log_sigma_5mm_3D_gldm_DependenceVariance	Laplacian	Texture Features--GLDM
log_sigma_5mm_3D_gldm_GrayLevelNonUniformity	Laplacian	Texture Features--GLDM
log_sigma_5mm_3D_gldm_GrayLevelVariance	Laplacian	Texture Features--GLDM
log_sigma_5mm_3D_gldm_HighGrayLevelEmphasis	Laplacian	Texture Features--GLDM
log_sigma_5mm_3D_gldm_LargeDependenceEmphasis	Laplacian	Texture Features--GLDM
log_sigma_5mm_3D_gldm_LargeDependenceHighGrayLevelEmphasis	Laplacian	Texture Features--GLDM
log_sigma_5mm_3D_gldm_LargeDependenceLowGrayLevelEmphasis	Laplacian	Texture Features--

		GLDM
log_sigma_5mm_3D_gldm_LowGrayLevelEmphasis	Laplacian	Texture Features--GLDM
log_sigma_5mm_3D_gldm_SmallDependenceEmphasis	Laplacian	Texture Features--GLDM
log_sigma_5mm_3D_gldm_SmallDependenceHighGrayLevelEmphasis	Laplacian	Texture Features--GLDM
log_sigma_5mm_3D_gldm_SmallDependenceLowGrayLevelEmphasis	Laplacian	Texture Features--GLDM
log_sigma_5mm_3D_ngtdm_Busyness	Laplacian	Texture Features--NGTDM
log_sigma_5mm_3D_ngtdm_Coarseness	Laplacian	Texture Features--NGTDM
log_sigma_5mm_3D_ngtdm_Complexity	Laplacian	Texture Features--NGTDM
log_sigma_5mm_3D_ngtdm_Contrast	Laplacian	Texture Features--NGTDM
log_sigma_5mm_3D_ngtdm_Strength	Laplacian	Texture Features--NGTDM
wavelet_LLH_firstorder_10Percentile	wavelet	First Order Features
wavelet_LLH_firstorder_90Percentile	wavelet	First Order Features
wavelet_LLH_firstorder_Energy	wavelet	First Order Features

wavelet_LLH_firstorder_Entropy	wavelet	First Order Features
wavelet_LLH_firstorder_InterquartileRange	wavelet	First Order Features
wavelet_LLH_firstorder_Kurtosis	wavelet	First Order Features
wavelet_LLH_firstorder_Maximum	wavelet	First Order Features
wavelet_LLH_firstorder_MeanAbsoluteDeviation	wavelet	First Order Features
wavelet_LLH_firstorder_Mean	wavelet	First Order Features
wavelet_LLH_firstorder_Median	wavelet	First Order Features
wavelet_LLH_firstorder_Minimum	wavelet	First Order Features
wavelet_LLH_firstorder_Range	wavelet	First Order Features
wavelet_LLH_firstorder_RobustMeanAbsoluteDeviation	wavelet	First Order Features
wavelet_LLH_firstorder_RootMeanSquared	wavelet	First Order Features
wavelet_LLH_firstorder_Skewness	wavelet	First Order Features
wavelet_LLH_firstorder_TotalEnergy	wavelet	First Order Features
wavelet_LLH_firstorder_Uniformity	wavelet	First Order Features
wavelet_LLH_firstorder_Variance	wavelet	First Order Features

wavelet_LLH_glcm_Autocorrelation	wavelet	Texture Features--GLCM
wavelet_LLH_glcm_JointAverage	wavelet	Texture Features--GLCM
wavelet_LLH_glcm_ClusterProminence	wavelet	Texture Features--GLCM
wavelet_LLH_glcm_ClusterShade	wavelet	Texture Features--GLCM
wavelet_LLH_glcm_ClusterTendency	wavelet	Texture Features--GLCM
wavelet_LLH_glcm_Contrast	wavelet	Texture Features--GLCM
wavelet_LLH_glcm_Correlation	wavelet	Texture Features--GLCM
wavelet_LLH_glcm_DifferenceAverage	wavelet	Texture Features--GLCM
wavelet_LLH_glcm_DifferenceEntropy	wavelet	Texture Features--GLCM
wavelet_LLH_glcm_DifferenceVariance	wavelet	Texture Features--GLCM
wavelet_LLH_glcm_JointEnergy	wavelet	Texture Features--GLCM
wavelet_LLH_glcm_JointEntropy	wavelet	Texture Features--GLCM
wavelet_LLH_glcm_Imc1	wavelet	Texture Features--GLCM
wavelet_LLH_glcm_Imc2	wavelet	Texture Features--GLCM
wavelet_LLH_glcm_Idm	wavelet	Texture Features--GLCM

wavelet_LLH_glcm_Idmn	wavelet	Texture Features--GLCM
wavelet_LLH_glcm_Id	wavelet	Texture Features--GLCM
wavelet_LLH_glcm_Idn	wavelet	Texture Features--GLCM
wavelet_LLH_glcm_InverseVariance	wavelet	Texture Features--GLCM
wavelet_LLH_glcm_MaximumProbability	wavelet	Texture Features--GLCM
wavelet_LLH_glcm_SumEntropy	wavelet	Texture Features--GLCM
wavelet_LLH_glcm_SumSquares	wavelet	Texture Features--GLCM
wavelet_LLH_glrlm_GrayLevelNonUniformity	wavelet	Texture Features--GLRLM
wavelet_LLH_glrlm_GrayLevelNonUniformityNormalized	wavelet	Texture Features--GLRLM
wavelet_LLH_glrlm_GrayLevelVariance	wavelet	Texture Features--GLRLM
wavelet_LLH_glrlm_HighGrayLevelRunEmphasis	wavelet	Texture Features--GLRLM
wavelet_LLH_glrlm_LongRunEmphasis	wavelet	Texture Features--GLRLM
wavelet_LLH_glrlm_LongRunHighGrayLevelEmphasis	wavelet	Texture Features--GLRLM

wavelet_LLH_glrlm_LongRunLowGrayLevelEmphasis	wavelet	Texture Features-- GLRLM
wavelet_LLH_glrlm_LowGrayLevelRunEmphasis	wavelet	Texture Features-- GLRLM
wavelet_LLH_glrlm_RunEntropy	wavelet	Texture Features-- GLRLM
wavelet_LLH_glrlm_RunLengthNonUniformity	wavelet	Texture Features-- GLRLM
wavelet_LLH_glrlm_RunLengthNonUniformityNormalized	wavelet	Texture Features-- GLRLM
wavelet_LLH_glrlm_RunPercentage	wavelet	Texture Features-- GLRLM
wavelet_LLH_glrlm_RunVariance	wavelet	Texture Features-- GLRLM
wavelet_LLH_glrlm_ShortRunEmphasis	wavelet	Texture Features-- GLRLM
wavelet_LLH_glrlm_ShortRunHighGrayLevelEmphasis	wavelet	Texture Features-- GLRLM
wavelet_LLH_glrlm_ShortRunLowGrayLevelEmphasis	wavelet	Texture Features-- GLRLM
wavelet_LLH_glszm_GrayLevelNonUniformity	wavelet	Texture Features-- GLSZM
wavelet_LLH_glszm_GrayLevelNonUniformityNormalized	wavelet	Texture Features-- GLSZM

wavelet_LLH_glszm_GrayLevelVariance	wavelet	Texture Features-- GLSZM
wavelet_LLH_glszm_HighGrayLevelZoneEmphasis	wavelet	Texture Features-- GLSZM
wavelet_LLH_glszm_LargeAreaEmphasis	wavelet	Texture Features-- GLSZM
wavelet_LLH_glszm_LargeAreaHighGrayLevelEmphasis	wavelet	Texture Features-- GLSZM
wavelet_LLH_glszm_LargeAreaLowGrayLevelEmphasis	wavelet	Texture Features-- GLSZM
wavelet_LLH_glszm_LowGrayLevelZoneEmphasis	wavelet	Texture Features-- GLSZM
wavelet_LLH_glszm_SizeZoneNonUniformity	wavelet	Texture Features-- GLSZM
wavelet_LLH_glszm_SizeZoneNonUniformityNormalized	wavelet	Texture Features-- GLSZM
wavelet_LLH_glszm_SmallAreaEmphasis	wavelet	Texture Features-- GLSZM
wavelet_LLH_glszm_SmallAreaHighGrayLevelEmphasis	wavelet	Texture Features-- GLSZM
wavelet_LLH_glszm_SmallAreaLowGrayLevelEmphasis	wavelet	Texture Features-- GLSZM
wavelet_LLH_glszm_ZoneEntropy	wavelet	Texture Features--

		GLSZM
wavelet_LLH_glszm_ZonePercentage	wavelet	Texture Features--GLSZM
wavelet_LLH_glszm_ZoneVariance	wavelet	Texture Features--GLSZM
wavelet_LLH_gldm_DependenceEntropy	wavelet	Texture Features--GLDM
wavelet_LLH_gldm_DependenceNonUniformity	wavelet	Texture Features--GLDM
wavelet_LLH_gldm_DependenceNonUniformityNormalized	wavelet	Texture Features--GLDM
wavelet_LLH_gldm_DependenceVariance	wavelet	Texture Features--GLDM
wavelet_LLH_gldm_GrayLevelNonUniformity	wavelet	Texture Features--GLDM
wavelet_LLH_gldm_GrayLevelVariance	wavelet	Texture Features--GLDM
wavelet_LLH_gldm_HighGrayLevelEmphasis	wavelet	Texture Features--GLDM
wavelet_LLH_gldm_LargeDependenceEmphasis	wavelet	Texture Features--GLDM
wavelet_LLH_gldm_LargeDependenceHighGrayLevelEmphasis	wavelet	Texture Features--GLDM
wavelet_LLH_gldm_LargeDependenceLowGrayLevelEmphasis	wavelet	Texture Features--

		GLDM
wavelet_LLH_gldm_LowGrayLevelEmphasis	wavelet	Texture Features--GLDM
wavelet_LLH_gldm_SmallDependenceEmphasis	wavelet	Texture Features--GLDM
wavelet_LLH_gldm_SmallDependenceHighGrayLevelEmphasis	wavelet	Texture Features--GLDM
wavelet_LLH_gldm_SmallDependenceLowGrayLevelEmphasis	wavelet	Texture Features--GLDM
wavelet_LLH_ngtdm_Busyness	wavelet	Texture Features--NGTDM
wavelet_LLH_ngtdm_Coarseness	wavelet	Texture Features--NGTDM
wavelet_LLH_ngtdm_Complexity	wavelet	Texture Features--NGTDM
wavelet_LLH_ngtdm_Contrast	wavelet	Texture Features--NGTDM
wavelet_LLH_ngtdm_Strength	wavelet	Texture Features--NGTDM
wavelet_LHL_firstorder_10Percentile	wavelet	First Order Features
wavelet_LHL_firstorder_90Percentile	wavelet	First Order Features
wavelet_LHL_firstorder_Energy	wavelet	First Order Features

wavelet_LHL_firstorder_Entropy	wavelet	First Order Features
wavelet_LHL_firstorder_InterquartileRange	wavelet	First Order Features
wavelet_LHL_firstorder_Kurtosis	wavelet	First Order Features
wavelet_LHL_firstorder_Maximum	wavelet	First Order Features
wavelet_LHL_firstorder_MeanAbsoluteDeviation	wavelet	First Order Features
wavelet_LHL_firstorder_Mean	wavelet	First Order Features
wavelet_LHL_firstorder_Median	wavelet	First Order Features
wavelet_LHL_firstorder_Minimum	wavelet	First Order Features
wavelet_LHL_firstorder_Range	wavelet	First Order Features
wavelet_LHL_firstorder_RobustMeanAbsoluteDeviation	wavelet	First Order Features
wavelet_LHL_firstorder_RootMeanSquared	wavelet	First Order Features
wavelet_LHL_firstorder_Skewness	wavelet	First Order Features
wavelet_LHL_firstorder_TotalEnergy	wavelet	First Order Features
wavelet_LHL_firstorder_Uniformity	wavelet	First Order Features
wavelet_LHL_firstorder_Variance	wavelet	First Order Features

wavelet_LHL_glcm_Autocorrelation	wavelet	Texture Features--GLCM
wavelet_LHL_glcm_JointAverage	wavelet	Texture Features--GLCM
wavelet_LHL_glcm_ClusterProminence	wavelet	Texture Features--GLCM
wavelet_LHL_glcm_ClusterShade	wavelet	Texture Features--GLCM
wavelet_LHL_glcm_ClusterTendency	wavelet	Texture Features--GLCM
wavelet_LHL_glcm_Contrast	wavelet	Texture Features--GLCM
wavelet_LHL_glcm_Correlation	wavelet	Texture Features--GLCM
wavelet_LHL_glcm_DifferenceAverage	wavelet	Texture Features--GLCM
wavelet_LHL_glcm_DifferenceEntropy	wavelet	Texture Features--GLCM
wavelet_LHL_glcm_DifferenceVariance	wavelet	Texture Features--GLCM
wavelet_LHL_glcm_JointEnergy	wavelet	Texture Features--GLCM
wavelet_LHL_glcm_JointEntropy	wavelet	Texture Features--GLCM
wavelet_LHL_glcm_Imc1	wavelet	Texture Features--GLCM
wavelet_LHL_glcm_Imc2	wavelet	Texture Features--GLCM
wavelet_LHL_glcm_Idm	wavelet	Texture Features--GLCM

wavelet_LHL_glcm_Idmn	wavelet	Texture Features--GLCM
wavelet_LHL_glcm_Id	wavelet	Texture Features--GLCM
wavelet_LHL_glcm_Idn	wavelet	Texture Features--GLCM
wavelet_LHL_glcm_InverseVariance	wavelet	Texture Features--GLCM
wavelet_LHL_glcm_MaximumProbability	wavelet	Texture Features--GLCM
wavelet_LHL_glcm_SumEntropy	wavelet	Texture Features--GLCM
wavelet_LHL_glcm_SumSquares	wavelet	Texture Features--GLCM
wavelet_LHL_glrlm_GrayLevelNonUniformity	wavelet	Texture Features--GLRLM
wavelet_LHL_glrlm_GrayLevelNonUniformityNormalized	wavelet	Texture Features--GLRLM
wavelet_LHL_glrlm_GrayLevelVariance	wavelet	Texture Features--GLRLM
wavelet_LHL_glrlm_HighGrayLevelRunEmphasis	wavelet	Texture Features--GLRLM
wavelet_LHL_glrlm_LongRunEmphasis	wavelet	Texture Features--GLRLM
wavelet_LHL_glrlm_LongRunHighGrayLevelEmphasis	wavelet	Texture Features--GLRLM
wavelet_LHL_glrlm_LongRunLowGrayLevelEmphasis	wavelet	Texture Features--

		GLRLM
wavelet_LHL_glrlm_LowGrayLevelRunEmphasis	wavelet	Texture Features--GLRLM
wavelet_LHL_glrlm_RunEntropy	wavelet	Texture Features--GLRLM
wavelet_LHL_glrlm_RunLengthNonUniformity	wavelet	Texture Features--GLRLM
wavelet_LHL_glrlm_RunLengthNonUniformityNormalized	wavelet	Texture Features--GLRLM
wavelet_LHL_glrlm_RunPercentage	wavelet	Texture Features--GLRLM
wavelet_LHL_glrlm_RunVariance	wavelet	Texture Features--GLRLM
wavelet_LHL_glrlm_ShortRunEmphasis	wavelet	Texture Features--GLRLM
wavelet_LHL_glrlm_ShortRunHighGrayLevelEmphasis	wavelet	Texture Features--GLRLM
wavelet_LHL_glrlm_ShortRunLowGrayLevelEmphasis	wavelet	Texture Features--GLRLM
wavelet_LHL_glszm_GrayLevelNonUniformity	wavelet	Texture Features--GLSZM
wavelet_LHL_glszm_GrayLevelNonUniformityNormalized	wavelet	Texture Features--GLSZM

wavelet_LHL_glszm_GrayLevelVariance	wavelet	Texture Features--GLSZM
wavelet_LHL_glszm_HighGrayLevelZoneEmphasis	wavelet	Texture Features--GLSZM
wavelet_LHL_glszm_LargeAreaEmphasis	wavelet	Texture Features--GLSZM
wavelet_LHL_glszm_LargeAreaHighGrayLevelEmphasis	wavelet	Texture Features--GLSZM
wavelet_LHL_glszm_LargeAreaLowGrayLevelEmphasis	wavelet	Texture Features--GLSZM
wavelet_LHL_glszm_LowGrayLevelZoneEmphasis	wavelet	Texture Features--GLSZM
wavelet_LHL_glszm_SizeZoneNonUniformity	wavelet	Texture Features--GLSZM
wavelet_LHL_glszm_SizeZoneNonUniformityNormalized	wavelet	Texture Features--GLSZM
wavelet_LHL_glszm_SmallAreaEmphasis	wavelet	Texture Features--GLSZM
wavelet_LHL_glszm_SmallAreaHighGrayLevelEmphasis	wavelet	Texture Features--GLSZM
wavelet_LHL_glszm_SmallAreaLowGrayLevelEmphasis	wavelet	Texture Features--GLSZM
wavelet_LHL_glszm_ZoneEntropy	wavelet	Texture Features--GLSZM

wavelet_LHL_glszm_ZonePercentage	wavelet	Texture Features--GLSZM
wavelet_LHL_glszm_ZoneVariance	wavelet	Texture Features--GLSZM
wavelet_LHL_gldm_DependenceEntropy	wavelet	Texture Features--GLDM
wavelet_LHL_gldm_DependenceNonUniformity	wavelet	Texture Features--GLDM
wavelet_LHL_gldm_DependenceNonUniformityNormalized	wavelet	Texture Features--GLDM
wavelet_LHL_gldm_DependenceVariance	wavelet	Texture Features--GLDM
wavelet_LHL_gldm_GrayLevelNonUniformity	wavelet	Texture Features--GLDM
wavelet_LHL_gldm_GrayLevelVariance	wavelet	Texture Features--GLDM
wavelet_LHL_gldm_HighGrayLevelEmphasis	wavelet	Texture Features--GLDM
wavelet_LHL_gldm_LargeDependenceEmphasis	wavelet	Texture Features--GLDM
wavelet_LHL_gldm_LargeDependenceHighGrayLevelEmphasis	wavelet	Texture Features--GLDM
wavelet_LHL_gldm_LargeDependenceLowGrayLevelEmphasis	wavelet	Texture Features--

		GLDM
wavelet_LHL_gldm_LowGrayLevelEmphasis	wavelet	Texture Features--GLDM
wavelet_LHL_gldm_SmallDependenceEmphasis	wavelet	Texture Features--GLDM
wavelet_LHL_gldm_SmallDependenceHighGrayLevelEmphasis	wavelet	Texture Features--GLDM
wavelet_LHL_gldm_SmallDependenceLowGrayLevelEmphasis	wavelet	Texture Features--GLDM
wavelet_LHL_ngtdm_Busyness	wavelet	Texture Features--NGTDM
wavelet_LHL_ngtdm_Coarseness	wavelet	Texture Features--NGTDM
wavelet_LHL_ngtdm_Complexity	wavelet	Texture Features--NGTDM
wavelet_LHL_ngtdm_Contrast	wavelet	Texture Features--NGTDM
wavelet_LHL_ngtdm_Strength	wavelet	Texture Features--NGTDM
wavelet_LHH_firstorder_10Percentile	wavelet	First Order Features
wavelet_LHH_firstorder_90Percentile	wavelet	First Order Features
wavelet_LHH_firstorder_Energy	wavelet	First Order Features

wavelet_LHH_firstorder_Entropy	wavelet	First Order Features
wavelet_LHH_firstorder_InterquartileRange	wavelet	First Order Features
wavelet_LHH_firstorder_Kurtosis	wavelet	First Order Features
wavelet_LHH_firstorder_Maximum	wavelet	First Order Features
wavelet_LHH_firstorder_MeanAbsoluteDeviation	wavelet	First Order Features
wavelet_LHH_firstorder_Mean	wavelet	First Order Features
wavelet_LHH_firstorder_Median	wavelet	First Order Features
wavelet_LHH_firstorder_Minimum	wavelet	First Order Features
wavelet_LHH_firstorder_Range	wavelet	First Order Features
wavelet_LHH_firstorder_RobustMeanAbsoluteDeviation	wavelet	First Order Features
wavelet_LHH_firstorder_RootMeanSquared	wavelet	First Order Features
wavelet_LHH_firstorder_Skewness	wavelet	First Order Features
wavelet_LHH_firstorder_TotalEnergy	wavelet	First Order Features
wavelet_LHH_firstorder_Uniformity	wavelet	First Order Features
wavelet_LHH_firstorder_Variance	wavelet	First Order Features

wavelet_LHH_glcm_Autocorrelation	wavelet	Texture Features--GLCM
wavelet_LHH_glcm_JointAverage	wavelet	Texture Features--GLCM
wavelet_LHH_glcm_ClusterProminence	wavelet	Texture Features--GLCM
wavelet_LHH_glcm_ClusterShade	wavelet	Texture Features--GLCM
wavelet_LHH_glcm_ClusterTendency	wavelet	Texture Features--GLCM
wavelet_LHH_glcm_Contrast	wavelet	Texture Features--GLCM
wavelet_LHH_glcm_Correlation	wavelet	Texture Features--GLCM
wavelet_LHH_glcm_DifferenceAverage	wavelet	Texture Features--GLCM
wavelet_LHH_glcm_DifferenceEntropy	wavelet	Texture Features--GLCM
wavelet_LHH_glcm_DifferenceVariance	wavelet	Texture Features--GLCM
wavelet_LHH_glcm_JointEnergy	wavelet	Texture Features--GLCM
wavelet_LHH_glcm_JointEntropy	wavelet	Texture Features--GLCM
wavelet_LHH_glcm_Imc1	wavelet	Texture Features--GLCM
wavelet_LHH_glcm_Imc2	wavelet	Texture Features--GLCM
wavelet_LHH_glcm_Idm	wavelet	Texture Features--GLCM

wavelet_LHH_glcm_Idmn	wavelet	Texture Features--GLCM
wavelet_LHH_glcm_Id	wavelet	Texture Features--GLCM
wavelet_LHH_glcm_Idn	wavelet	Texture Features--GLCM
wavelet_LHH_glcm_InverseVariance	wavelet	Texture Features--GLCM
wavelet_LHH_glcm_MaximumProbability	wavelet	Texture Features--GLCM
wavelet_LHH_glcm_SumEntropy	wavelet	Texture Features--GLCM
wavelet_LHH_glcm_SumSquares	wavelet	Texture Features--GLCM
wavelet_LHH_glrlm_GrayLevelNonUniformity	wavelet	Texture Features--GLRLM
wavelet_LHH_glrlm_GrayLevelNonUniformityNormalized	wavelet	Texture Features--GLRLM
wavelet_LHH_glrlm_GrayLevelVariance	wavelet	Texture Features--GLRLM
wavelet_LHH_glrlm_HighGrayLevelRunEmphasis	wavelet	Texture Features--GLRLM
wavelet_LHH_glrlm_LongRunEmphasis	wavelet	Texture Features--GLRLM
wavelet_LHH_glrlm_LongRunHighGrayLevelEmphasis	wavelet	Texture Features--GLRLM

wavelet_LHH_glrlm_LongRunLowGrayLevelEmphasis	wavelet	Texture Features--GLRLM
wavelet_LHH_glrlm_LowGrayLevelRunEmphasis	wavelet	Texture Features--GLRLM
wavelet_LHH_glrlm_RunEntropy	wavelet	Texture Features--GLRLM
wavelet_LHH_glrlm_RunLengthNonUniformity	wavelet	Texture Features--GLRLM
wavelet_LHH_glrlm_RunLengthNonUniformityNormalized	wavelet	Texture Features--GLRLM
wavelet_LHH_glrlm_RunPercentage	wavelet	Texture Features--GLRLM
wavelet_LHH_glrlm_RunVariance	wavelet	Texture Features--GLRLM
wavelet_LHH_glrlm_ShortRunEmphasis	wavelet	Texture Features--GLRLM
wavelet_LHH_glrlm_ShortRunHighGrayLevelEmphasis	wavelet	Texture Features--GLRLM
wavelet_LHH_glrlm_ShortRunLowGrayLevelEmphasis	wavelet	Texture Features--GLRLM
wavelet_LHH_glszm_GrayLevelNonUniformity	wavelet	Texture Features--GLSZM
wavelet_LHH_glszm_GrayLevelNonUniformityNormalized	wavelet	Texture Features--GLSZM

wavelet_LHH_glszm_GrayLevelVariance	wavelet	Texture Features-- GLSZM
wavelet_LHH_glszm_HighGrayLevelZoneEmphasis	wavelet	Texture Features-- GLSZM
wavelet_LHH_glszm_LargeAreaEmphasis	wavelet	Texture Features-- GLSZM
wavelet_LHH_glszm_LargeAreaHighGrayLevelEmphasis	wavelet	Texture Features-- GLSZM
wavelet_LHH_glszm_LargeAreaLowGrayLevelEmphasis	wavelet	Texture Features-- GLSZM
wavelet_LHH_glszm_LowGrayLevelZoneEmphasis	wavelet	Texture Features-- GLSZM
wavelet_LHH_glszm_SizeZoneNonUniformity	wavelet	Texture Features-- GLSZM
wavelet_LHH_glszm_SizeZoneNonUniformityNormalized	wavelet	Texture Features-- GLSZM
wavelet_LHH_glszm_SmallAreaEmphasis	wavelet	Texture Features-- GLSZM
wavelet_LHH_glszm_SmallAreaHighGrayLevelEmphasis	wavelet	Texture Features-- GLSZM
wavelet_LHH_glszm_SmallAreaLowGrayLevelEmphasis	wavelet	Texture Features-- GLSZM
wavelet_LHH_glszm_ZoneEntropy	wavelet	Texture Features--

		GLSZM
wavelet_LHH_glszm_ZonePercentage	wavelet	Texture Features-- GLSZM
wavelet_LHH_glszm_ZoneVariance	wavelet	Texture Features-- GLSZM
wavelet_LHH_gldm_DependenceEntropy	wavelet	Texture Features-- GLDM
wavelet_LHH_gldm_DependenceNonUniformity	wavelet	Texture Features-- GLDM
wavelet_LHH_gldm_DependenceNonUniformityNormalized	wavelet	Texture Features-- GLDM
wavelet_LHH_gldm_DependenceVariance	wavelet	Texture Features-- GLDM
wavelet_LHH_gldm_GrayLevelNonUniformity	wavelet	Texture Features-- GLDM
wavelet_LHH_gldm_GrayLevelVariance	wavelet	Texture Features-- GLDM
wavelet_LHH_gldm_HighGrayLevelEmphasis	wavelet	Texture Features-- GLDM
wavelet_LHH_gldm_LargeDependenceEmphasis	wavelet	Texture Features-- GLDM
wavelet_LHH_gldm_LargeDependenceHighGrayLevelEmphasis	wavelet	Texture Features-- GLDM
wavelet_LHH_gldm_LargeDependenceLowGrayLevelEmphasis	wavelet	Texture Features--

		GLDM
wavelet_LHH_gldm_LowGrayLevelEmphasis	wavelet	Texture Features--GLDM
wavelet_LHH_gldm_SmallDependenceEmphasis	wavelet	Texture Features--GLDM
wavelet_LHH_gldm_SmallDependenceHighGrayLevelEmphasis	wavelet	Texture Features--GLDM
wavelet_LHH_gldm_SmallDependenceLowGrayLevelEmphasis	wavelet	Texture Features--GLDM
wavelet_LHH_ngtdm_Busyness	wavelet	Texture Features--NGTDM
wavelet_LHH_ngtdm_Coarseness	wavelet	Texture Features--NGTDM
wavelet_LHH_ngtdm_Complexity	wavelet	Texture Features--NGTDM
wavelet_LHH_ngtdm_Contrast	wavelet	Texture Features--NGTDM
wavelet_LHH_ngtdm_Strength	wavelet	Texture Features--NGTDM
wavelet_HLL_firstorder_10Percentile	wavelet	First Order Features
wavelet_HLL_firstorder_90Percentile	wavelet	First Order Features
wavelet_HLL_firstorder_Energy	wavelet	First Order Features

wavelet_HLL_firstorder_Entropy	wavelet	First Order Features
wavelet_HLL_firstorder_InterquartileRange	wavelet	First Order Features
wavelet_HLL_firstorder_Kurtosis	wavelet	First Order Features
wavelet_HLL_firstorder_Maximum	wavelet	First Order Features
wavelet_HLL_firstorder_MeanAbsoluteDeviation	wavelet	First Order Features
wavelet_HLL_firstorder_Mean	wavelet	First Order Features
wavelet_HLL_firstorder_Median	wavelet	First Order Features
wavelet_HLL_firstorder_Minimum	wavelet	First Order Features
wavelet_HLL_firstorder_Range	wavelet	First Order Features
wavelet_HLL_firstorder_RobustMeanAbsoluteDeviation	wavelet	First Order Features
wavelet_HLL_firstorder_RootMeanSquared	wavelet	First Order Features
wavelet_HLL_firstorder_Skewness	wavelet	First Order Features
wavelet_HLL_firstorder_TotalEnergy	wavelet	First Order Features
wavelet_HLL_firstorder_Uniformity	wavelet	First Order Features
wavelet_HLL_firstorder_Variance	wavelet	First Order Features

wavelet_HLL_glcm_Autocorrelation	wavelet	Texture Features--GLCM
wavelet_HLL_glcm_JointAverage	wavelet	Texture Features--GLCM
wavelet_HLL_glcm_ClusterProminence	wavelet	Texture Features--GLCM
wavelet_HLL_glcm_ClusterShade	wavelet	Texture Features--GLCM
wavelet_HLL_glcm_ClusterTendency	wavelet	Texture Features--GLCM
wavelet_HLL_glcm_Contrast	wavelet	Texture Features--GLCM
wavelet_HLL_glcm_Correlation	wavelet	Texture Features--GLCM
wavelet_HLL_glcm_DifferenceAverage	wavelet	Texture Features--GLCM
wavelet_HLL_glcm_DifferenceEntropy	wavelet	Texture Features--GLCM
wavelet_HLL_glcm_DifferenceVariance	wavelet	Texture Features--GLCM
wavelet_HLL_glcm_JointEnergy	wavelet	Texture Features--GLCM
wavelet_HLL_glcm_JointEntropy	wavelet	Texture Features--GLCM
wavelet_HLL_glcm_Imc1	wavelet	Texture Features--GLCM
wavelet_HLL_glcm_Imc2	wavelet	Texture Features--GLCM
wavelet_HLL_glcm_Idm	wavelet	Texture Features--GLCM

wavelet_HLL_glcm_Idmn	wavelet	Texture Features--GLCM
wavelet_HLL_glcm_Id	wavelet	Texture Features--GLCM
wavelet_HLL_glcm_Idn	wavelet	Texture Features--GLCM
wavelet_HLL_glcm_InverseVariance	wavelet	Texture Features--GLCM
wavelet_HLL_glcm_MaximumProbability	wavelet	Texture Features--GLCM
wavelet_HLL_glcm_SumEntropy	wavelet	Texture Features--GLCM
wavelet_HLL_glcm_SumSquares	wavelet	Texture Features--GLCM
wavelet_HLL_glrlm_GrayLevelNonUniformity	wavelet	Texture Features--GLRLM
wavelet_HLL_glrlm_GrayLevelNonUniformityNormalized	wavelet	Texture Features--GLRLM
wavelet_HLL_glrlm_GrayLevelVariance	wavelet	Texture Features--GLRLM
wavelet_HLL_glrlm_HighGrayLevelRunEmphasis	wavelet	Texture Features--GLRLM
wavelet_HLL_glrlm_LongRunEmphasis	wavelet	Texture Features--GLRLM
wavelet_HLL_glrlm_LongRunHighGrayLevelEmphasis	wavelet	Texture Features--GLRLM
wavelet_HLL_glrlm_LongRunLowGrayLevelEmphasis	wavelet	Texture Features--

		GLRLM
wavelet_HLL_glrlm_LowGrayLevelRunEmphasis	wavelet	Texture Features-- GLRLM
wavelet_HLL_glrlm_RunEntropy	wavelet	Texture Features-- GLRLM
wavelet_HLL_glrlm_RunLengthNonUniformity	wavelet	Texture Features-- GLRLM
wavelet_HLL_glrlm_RunLengthNonUniformityNormalized	wavelet	Texture Features-- GLRLM
wavelet_HLL_glrlm_RunPercentage	wavelet	Texture Features-- GLRLM
wavelet_HLL_glrlm_RunVariance	wavelet	Texture Features-- GLRLM
wavelet_HLL_glrlm_ShortRunEmphasis	wavelet	Texture Features-- GLRLM
wavelet_HLL_glrlm_ShortRunHighGrayLevelEmphasis	wavelet	Texture Features-- GLRLM
wavelet_HLL_glrlm_ShortRunLowGrayLevelEmphasis	wavelet	Texture Features-- GLRLM
wavelet_HLL_glszm_GrayLevelNonUniformity	wavelet	Texture Features-- GLSZM
wavelet_HLL_glszm_GrayLevelNonUniformityNormalized	wavelet	Texture Features-- GLSZM

wavelet_HLL_glszm_GrayLevelVariance	wavelet	Texture Features--GLSZM
wavelet_HLL_glszm_HighGrayLevelZoneEmphasis	wavelet	Texture Features--GLSZM
wavelet_HLL_glszm_LargeAreaEmphasis	wavelet	Texture Features--GLSZM
wavelet_HLL_glszm_LargeAreaHighGrayLevelEmphasis	wavelet	Texture Features--GLSZM
wavelet_HLL_glszm_LargeAreaLowGrayLevelEmphasis	wavelet	Texture Features--GLSZM
wavelet_HLL_glszm_LowGrayLevelZoneEmphasis	wavelet	Texture Features--GLSZM
wavelet_HLL_glszm_SizeZoneNonUniformity	wavelet	Texture Features--GLSZM
wavelet_HLL_glszm_SizeZoneNonUniformityNormalized	wavelet	Texture Features--GLSZM
wavelet_HLL_glszm_SmallAreaEmphasis	wavelet	Texture Features--GLSZM
wavelet_HLL_glszm_SmallAreaHighGrayLevelEmphasis	wavelet	Texture Features--GLSZM
wavelet_HLL_glszm_SmallAreaLowGrayLevelEmphasis	wavelet	Texture Features--GLSZM
wavelet_HLL_glszm_ZoneEntropy	wavelet	Texture Features--GLSZM

wavelet_HLL_glszm_ZonePercentage	wavelet	Texture Features--GLSZM
wavelet_HLL_glszm_ZoneVariance	wavelet	Texture Features--GLSZM
wavelet_HLL_gldm_DependenceEntropy	wavelet	Texture Features--GLDM
wavelet_HLL_gldm_DependenceNonUniformity	wavelet	Texture Features--GLDM
wavelet_HLL_gldm_DependenceNonUniformityNormalized	wavelet	Texture Features--GLDM
wavelet_HLL_gldm_DependenceVariance	wavelet	Texture Features--GLDM
wavelet_HLL_gldm_GrayLevelNonUniformity	wavelet	Texture Features--GLDM
wavelet_HLL_gldm_GrayLevelVariance	wavelet	Texture Features--GLDM
wavelet_HLL_gldm_HighGrayLevelEmphasis	wavelet	Texture Features--GLDM
wavelet_HLL_gldm_LargeDependenceEmphasis	wavelet	Texture Features--GLDM
wavelet_HLL_gldm_LargeDependenceHighGrayLevelEmphasis	wavelet	Texture Features--GLDM
wavelet_HLL_gldm_LargeDependenceLowGrayLevelEmphasis	wavelet	Texture Features--

		GLDM
wavelet_HLL_gldm_LowGrayLevelEmphasis	wavelet	Texture Features-- GLDM
wavelet_HLL_gldm_SmallDependenceEmphasis	wavelet	Texture Features-- GLDM
wavelet_HLL_gldm_SmallDependenceHighGrayLevelEmphasis	wavelet	Texture Features-- GLDM
wavelet_HLL_gldm_SmallDependenceLowGrayLevelEmphasis	wavelet	Texture Features-- GLDM
wavelet_HLL_ngtdm_Busyness	wavelet	Texture Features-- NGTDM
wavelet_HLL_ngtdm_Coarseness	wavelet	Texture Features-- NGTDM
wavelet_HLL_ngtdm_Complexity	wavelet	Texture Features-- NGTDM
wavelet_HLL_ngtdm_Contrast	wavelet	Texture Features-- NGTDM
wavelet_HLL_ngtdm_Strength	wavelet	Texture Features-- NGTDM
wavelet_HLH_firstorder_10Percentile	wavelet	First Order Features
wavelet_HLH_firstorder_90Percentile	wavelet	First Order Features
wavelet_HLH_firstorder_Energy	wavelet	First Order Features

wavelet_HLH_firstorder_Entropy	wavelet	First Order Features
wavelet_HLH_firstorder_InterquartileRange	wavelet	First Order Features
wavelet_HLH_firstorder_Kurtosis	wavelet	First Order Features
wavelet_HLH_firstorder_Maximum	wavelet	First Order Features
wavelet_HLH_firstorder_MeanAbsoluteDeviation	wavelet	First Order Features
wavelet_HLH_firstorder_Mean	wavelet	First Order Features
wavelet_HLH_firstorder_Median	wavelet	First Order Features
wavelet_HLH_firstorder_Minimum	wavelet	First Order Features
wavelet_HLH_firstorder_Range	wavelet	First Order Features
wavelet_HLH_firstorder_RobustMeanAbsoluteDeviation	wavelet	First Order Features
wavelet_HLH_firstorder_RootMeanSquared	wavelet	First Order Features
wavelet_HLH_firstorder_Skewness	wavelet	First Order Features
wavelet_HLH_firstorder_TotalEnergy	wavelet	First Order Features
wavelet_HLH_firstorder_Uniformity	wavelet	First Order Features
wavelet_HLH_firstorder_Variance	wavelet	First Order Features

wavelet_HLH_glcm_Autocorrelation	wavelet	Texture Features--GLCM
wavelet_HLH_glcm_JointAverage	wavelet	Texture Features--GLCM
wavelet_HLH_glcm_ClusterProminence	wavelet	Texture Features--GLCM
wavelet_HLH_glcm_ClusterShade	wavelet	Texture Features--GLCM
wavelet_HLH_glcm_ClusterTendency	wavelet	Texture Features--GLCM
wavelet_HLH_glcm_Contrast	wavelet	Texture Features--GLCM
wavelet_HLH_glcm_Correlation	wavelet	Texture Features--GLCM
wavelet_HLH_glcm_DifferenceAverage	wavelet	Texture Features--GLCM
wavelet_HLH_glcm_DifferenceEntropy	wavelet	Texture Features--GLCM
wavelet_HLH_glcm_DifferenceVariance	wavelet	Texture Features--GLCM
wavelet_HLH_glcm_JointEnergy	wavelet	Texture Features--GLCM
wavelet_HLH_glcm_JointEntropy	wavelet	Texture Features--GLCM
wavelet_HLH_glcm_Imc1	wavelet	Texture Features--GLCM
wavelet_HLH_glcm_Imc2	wavelet	Texture Features--GLCM
wavelet_HLH_glcm_Idm	wavelet	Texture Features--GLCM

wavelet_HLH_glcm_Idmn	wavelet	Texture Features--GLCM
wavelet_HLH_glcm_Id	wavelet	Texture Features--GLCM
wavelet_HLH_glcm_Idn	wavelet	Texture Features--GLCM
wavelet_HLH_glcm_InverseVariance	wavelet	Texture Features--GLCM
wavelet_HLH_glcm_MaximumProbability	wavelet	Texture Features--GLCM
wavelet_HLH_glcm_SumEntropy	wavelet	Texture Features--GLCM
wavelet_HLH_glcm_SumSquares	wavelet	Texture Features--GLCM
wavelet_HLH_glrlm_GrayLevelNonUniformity	wavelet	Texture Features--GLRLM
wavelet_HLH_glrlm_GrayLevelNonUniformityNormalized	wavelet	Texture Features--GLRLM
wavelet_HLH_glrlm_GrayLevelVariance	wavelet	Texture Features--GLRLM
wavelet_HLH_glrlm_HighGrayLevelRunEmphasis	wavelet	Texture Features--GLRLM
wavelet_HLH_glrlm_LongRunEmphasis	wavelet	Texture Features--GLRLM
wavelet_HLH_glrlm_LongRunHighGrayLevelEmphasis	wavelet	Texture Features--GLRLM

wavelet_HLH_glrlm_LongRunLowGrayLevelEmphasis	wavelet	Texture Features--GLRLM
wavelet_HLH_glrlm_LowGrayLevelRunEmphasis	wavelet	Texture Features--GLRLM
wavelet_HLH_glrlm_RunEntropy	wavelet	Texture Features--GLRLM
wavelet_HLH_glrlm_RunLengthNonUniformity	wavelet	Texture Features--GLRLM
wavelet_HLH_glrlm_RunLengthNonUniformityNormalized	wavelet	Texture Features--GLRLM
wavelet_HLH_glrlm_RunPercentage	wavelet	Texture Features--GLRLM
wavelet_HLH_glrlm_RunVariance	wavelet	Texture Features--GLRLM
wavelet_HLH_glrlm_ShortRunEmphasis	wavelet	Texture Features--GLRLM
wavelet_HLH_glrlm_ShortRunHighGrayLevelEmphasis	wavelet	Texture Features--GLRLM
wavelet_HLH_glrlm_ShortRunLowGrayLevelEmphasis	wavelet	Texture Features--GLRLM
wavelet_HLH_glszm_GrayLevelNonUniformity	wavelet	Texture Features--GLSZM
wavelet_HLH_glszm_GrayLevelNonUniformityNormalized	wavelet	Texture Features--GLSZM

wavelet_HLH_glszm_GrayLevelVariance	wavelet	Texture Features-- GLSZM
wavelet_HLH_glszm_HighGrayLevelZoneEmphasis	wavelet	Texture Features-- GLSZM
wavelet_HLH_glszm_LargeAreaEmphasis	wavelet	Texture Features-- GLSZM
wavelet_HLH_glszm_LargeAreaHighGrayLevelEmphasis	wavelet	Texture Features-- GLSZM
wavelet_HLH_glszm_LargeAreaLowGrayLevelEmphasis	wavelet	Texture Features-- GLSZM
wavelet_HLH_glszm_LowGrayLevelZoneEmphasis	wavelet	Texture Features-- GLSZM
wavelet_HLH_glszm_SizeZoneNonUniformity	wavelet	Texture Features-- GLSZM
wavelet_HLH_glszm_SizeZoneNonUniformityNormalized	wavelet	Texture Features-- GLSZM
wavelet_HLH_glszm_SmallAreaEmphasis	wavelet	Texture Features-- GLSZM
wavelet_HLH_glszm_SmallAreaHighGrayLevelEmphasis	wavelet	Texture Features-- GLSZM
wavelet_HLH_glszm_SmallAreaLowGrayLevelEmphasis	wavelet	Texture Features-- GLSZM
wavelet_HLH_glszm_ZoneEntropy	wavelet	Texture Features--

		GLSZM
wavelet_HLH_glszm_ZonePercentage	wavelet	Texture Features--GLSZM
wavelet_HLH_glszm_ZoneVariance	wavelet	Texture Features--GLSZM
wavelet_HLH_gldm_DependenceEntropy	wavelet	Texture Features--GLDM
wavelet_HLH_gldm_DependenceNonUniformity	wavelet	Texture Features--GLDM
wavelet_HLH_gldm_DependenceNonUniformityNormalized	wavelet	Texture Features--GLDM
wavelet_HLH_gldm_DependenceVariance	wavelet	Texture Features--GLDM
wavelet_HLH_gldm_GrayLevelNonUniformity	wavelet	Texture Features--GLDM
wavelet_HLH_gldm_GrayLevelVariance	wavelet	Texture Features--GLDM
wavelet_HLH_gldm_HighGrayLevelEmphasis	wavelet	Texture Features--GLDM
wavelet_HLH_gldm_LargeDependenceEmphasis	wavelet	Texture Features--GLDM
wavelet_HLH_gldm_LargeDependenceHighGrayLevelEmphasis	wavelet	Texture Features--GLDM
wavelet_HLH_gldm_LargeDependenceLowGrayLevelEmphasis	wavelet	Texture Features--

		GLDM
wavelet_HLH_gldm_LowGrayLevelEmphasis	wavelet	Texture Features--GLDM
wavelet_HLH_gldm_SmallDependenceEmphasis	wavelet	Texture Features--GLDM
wavelet_HLH_gldm_SmallDependenceHighGrayLevelEmphasis	wavelet	Texture Features--GLDM
wavelet_HLH_gldm_SmallDependenceLowGrayLevelEmphasis	wavelet	Texture Features--GLDM
wavelet_HLH_ngtdm_Busyness	wavelet	Texture Features--NGTDM
wavelet_HLH_ngtdm_Coarseness	wavelet	Texture Features--NGTDM
wavelet_HLH_ngtdm_Complexity	wavelet	Texture Features--NGTDM
wavelet_HLH_ngtdm_Contrast	wavelet	Texture Features--NGTDM
wavelet_HLH_ngtdm_Strength	wavelet	Texture Features--NGTDM
wavelet_HHL_firstorder_10Percentile	wavelet	First Order Features
wavelet_HHL_firstorder_90Percentile	wavelet	First Order Features
wavelet_HHL_firstorder_Energy	wavelet	First Order Features

wavelet_HHL_firstorder_Entropy	wavelet	First Order Features
wavelet_HHL_firstorder_InterquartileRange	wavelet	First Order Features
wavelet_HHL_firstorder_Kurtosis	wavelet	First Order Features
wavelet_HHL_firstorder_Maximum	wavelet	First Order Features
wavelet_HHL_firstorder_MeanAbsoluteDeviation	wavelet	First Order Features
wavelet_HHL_firstorder_Mean	wavelet	First Order Features
wavelet_HHL_firstorder_Median	wavelet	First Order Features
wavelet_HHL_firstorder_Minimum	wavelet	First Order Features
wavelet_HHL_firstorder_Range	wavelet	First Order Features
wavelet_HHL_firstorder_RobustMeanAbsoluteDeviation	wavelet	First Order Features
wavelet_HHL_firstorder_RootMeanSquared	wavelet	First Order Features
wavelet_HHL_firstorder_Skewness	wavelet	First Order Features
wavelet_HHL_firstorder_TotalEnergy	wavelet	First Order Features
wavelet_HHL_firstorder_Uniformity	wavelet	First Order Features
wavelet_HHL_firstorder_Variance	wavelet	First Order Features

wavelet_HHL_glcm_Autocorrelation	wavelet	Texture Features--GLCM
wavelet_HHL_glcm_JointAverage	wavelet	Texture Features--GLCM
wavelet_HHL_glcm_ClusterProminence	wavelet	Texture Features--GLCM
wavelet_HHL_glcm_ClusterShade	wavelet	Texture Features--GLCM
wavelet_HHL_glcm_ClusterTendency	wavelet	Texture Features--GLCM
wavelet_HHL_glcm_Contrast	wavelet	Texture Features--GLCM
wavelet_HHL_glcm_Correlation	wavelet	Texture Features--GLCM
wavelet_HHL_glcm_DifferenceAverage	wavelet	Texture Features--GLCM
wavelet_HHL_glcm_DifferenceEntropy	wavelet	Texture Features--GLCM
wavelet_HHL_glcm_DifferenceVariance	wavelet	Texture Features--GLCM
wavelet_HHL_glcm_JointEnergy	wavelet	Texture Features--GLCM
wavelet_HHL_glcm_JointEntropy	wavelet	Texture Features--GLCM
wavelet_HHL_glcm_Imc1	wavelet	Texture Features--GLCM
wavelet_HHL_glcm_Imc2	wavelet	Texture Features--GLCM
wavelet_HHL_glcm_Idm	wavelet	Texture Features--GLCM

wavelet_HHL_glcm_Idmn	wavelet	Texture Features--GLCM
wavelet_HHL_glcm_Id	wavelet	Texture Features--GLCM
wavelet_HHL_glcm_Idn	wavelet	Texture Features--GLCM
wavelet_HHL_glcm_InverseVariance	wavelet	Texture Features--GLCM
wavelet_HHL_glcm_MaximumProbability	wavelet	Texture Features--GLCM
wavelet_HHL_glcm_SumEntropy	wavelet	Texture Features--GLCM
wavelet_HHL_glcm_SumSquares	wavelet	Texture Features--GLCM
wavelet_HHL_glrlm_GrayLevelNonUniformity	wavelet	Texture Features--GLRLM
wavelet_HHL_glrlm_GrayLevelNonUniformityNormalized	wavelet	Texture Features--GLRLM
wavelet_HHL_glrlm_GrayLevelVariance	wavelet	Texture Features--GLRLM
wavelet_HHL_glrlm_HighGrayLevelRunEmphasis	wavelet	Texture Features--GLRLM
wavelet_HHL_glrlm_LongRunEmphasis	wavelet	Texture Features--GLRLM
wavelet_HHL_glrlm_LongRunHighGrayLevelEmphasis	wavelet	Texture Features--GLRLM
wavelet_HHL_glrlm_LongRunLowGrayLevelEmphasis	wavelet	Texture Features--

		GLRLM
wavelet_HHL_glrlm_LowGrayLevelRunEmphasis	wavelet	Texture Features--GLRLM
wavelet_HHL_glrlm_RunEntropy	wavelet	Texture Features--GLRLM
wavelet_HHL_glrlm_RunLengthNonUniformity	wavelet	Texture Features--GLRLM
wavelet_HHL_glrlm_RunLengthNonUniformityNormalized	wavelet	Texture Features--GLRLM
wavelet_HHL_glrlm_RunPercentage	wavelet	Texture Features--GLRLM
wavelet_HHL_glrlm_RunVariance	wavelet	Texture Features--GLRLM
wavelet_HHL_glrlm_ShortRunEmphasis	wavelet	Texture Features--GLRLM
wavelet_HHL_glrlm_ShortRunHighGrayLevelEmphasis	wavelet	Texture Features--GLRLM
wavelet_HHL_glrlm_ShortRunLowGrayLevelEmphasis	wavelet	Texture Features--GLRLM
wavelet_HHL_glszm_GrayLevelNonUniformity	wavelet	Texture Features--GLSZM
wavelet_HHL_glszm_GrayLevelNonUniformityNormalized	wavelet	Texture Features--GLSZM

wavelet_HHL_glszm_GrayLevelVariance	wavelet	Texture Features--GLSZM
wavelet_HHL_glszm_HighGrayLevelZoneEmphasis	wavelet	Texture Features--GLSZM
wavelet_HHL_glszm_LargeAreaEmphasis	wavelet	Texture Features--GLSZM
wavelet_HHL_glszm_LargeAreaHighGrayLevelEmphasis	wavelet	Texture Features--GLSZM
wavelet_HHL_glszm_LargeAreaLowGrayLevelEmphasis	wavelet	Texture Features--GLSZM
wavelet_HHL_glszm_LowGrayLevelZoneEmphasis	wavelet	Texture Features--GLSZM
wavelet_HHL_glszm_SizeZoneNonUniformity	wavelet	Texture Features--GLSZM
wavelet_HHL_glszm_SizeZoneNonUniformityNormalized	wavelet	Texture Features--GLSZM
wavelet_HHL_glszm_SmallAreaEmphasis	wavelet	Texture Features--GLSZM
wavelet_HHL_glszm_SmallAreaHighGrayLevelEmphasis	wavelet	Texture Features--GLSZM
wavelet_HHL_glszm_SmallAreaLowGrayLevelEmphasis	wavelet	Texture Features--GLSZM
wavelet_HHL_glszm_ZoneEntropy	wavelet	Texture Features--GLSZM

wavelet_HHL_glszm_ZonePercentage	wavelet	Texture Features--GLSZM
wavelet_HHL_glszm_ZoneVariance	wavelet	Texture Features--GLSZM
wavelet_HHL_gldm_DependenceEntropy	wavelet	Texture Features--GLDM
wavelet_HHL_gldm_DependenceNonUniformity	wavelet	Texture Features--GLDM
wavelet_HHL_gldm_DependenceNonUniformityNormalized	wavelet	Texture Features--GLDM
wavelet_HHL_gldm_DependenceVariance	wavelet	Texture Features--GLDM
wavelet_HHL_gldm_GrayLevelNonUniformity	wavelet	Texture Features--GLDM
wavelet_HHL_gldm_GrayLevelVariance	wavelet	Texture Features--GLDM
wavelet_HHL_gldm_HighGrayLevelEmphasis	wavelet	Texture Features--GLDM
wavelet_HHL_gldm_LargeDependenceEmphasis	wavelet	Texture Features--GLDM
wavelet_HHL_gldm_LargeDependenceHighGrayLevelEmphasis	wavelet	Texture Features--GLDM
wavelet_HHL_gldm_LargeDependenceLowGrayLevelEmphasis	wavelet	Texture Features--

		GLDM
wavelet_HHL_gldm_LowGrayLevelEmphasis	wavelet	Texture Features--GLDM
wavelet_HHL_gldm_SmallDependenceEmphasis	wavelet	Texture Features--GLDM
wavelet_HHL_gldm_SmallDependenceHighGrayLevelEmphasis	wavelet	Texture Features--GLDM
wavelet_HHL_gldm_SmallDependenceLowGrayLevelEmphasis	wavelet	Texture Features--GLDM
wavelet_HHL_ngtdm_Busyness	wavelet	Texture Features--NGTDM
wavelet_HHL_ngtdm_Coarseness	wavelet	Texture Features--NGTDM
wavelet_HHL_ngtdm_Complexity	wavelet	Texture Features--NGTDM
wavelet_HHL_ngtdm_Contrast	wavelet	Texture Features--NGTDM
wavelet_HHL_ngtdm_Strength	wavelet	Texture Features--NGTDM
wavelet_HHH_firstorder_10Percentile	wavelet	First Order Features
wavelet_HHH_firstorder_90Percentile	wavelet	First Order Features
wavelet_HHH_firstorder_Energy	wavelet	First Order Features

wavelet_HHH_firstorder_Entropy	wavelet	First Order Features
wavelet_HHH_firstorder_InterquartileRange	wavelet	First Order Features
wavelet_HHH_firstorder_Kurtosis	wavelet	First Order Features
wavelet_HHH_firstorder_Maximum	wavelet	First Order Features
wavelet_HHH_firstorder_MeanAbsoluteDeviation	wavelet	First Order Features
wavelet_HHH_firstorder_Mean	wavelet	First Order Features
wavelet_HHH_firstorder_Median	wavelet	First Order Features
wavelet_HHH_firstorder_Minimum	wavelet	First Order Features
wavelet_HHH_firstorder_Range	wavelet	First Order Features
wavelet_HHH_firstorder_RobustMeanAbsoluteDeviation	wavelet	First Order Features
wavelet_HHH_firstorder_RootMeanSquared	wavelet	First Order Features
wavelet_HHH_firstorder_Skewness	wavelet	First Order Features
wavelet_HHH_firstorder_TotalEnergy	wavelet	First Order Features
wavelet_HHH_firstorder_Uniformity	wavelet	First Order Features
wavelet_HHH_firstorder_Variance	wavelet	First Order Features

wavelet_HHH_glcm_Autocorrelation	wavelet	Texture Features--GLCM
wavelet_HHH_glcm_JointAverage	wavelet	Texture Features--GLCM
wavelet_HHH_glcm_ClusterProminence	wavelet	Texture Features--GLCM
wavelet_HHH_glcm_ClusterShade	wavelet	Texture Features--GLCM
wavelet_HHH_glcm_ClusterTendency	wavelet	Texture Features--GLCM
wavelet_HHH_glcm_Contrast	wavelet	Texture Features--GLCM
wavelet_HHH_glcm_Correlation	wavelet	Texture Features--GLCM
wavelet_HHH_glcm_DifferenceAverage	wavelet	Texture Features--GLCM
wavelet_HHH_glcm_DifferenceEntropy	wavelet	Texture Features--GLCM
wavelet_HHH_glcm_DifferenceVariance	wavelet	Texture Features--GLCM
wavelet_HHH_glcm_JointEnergy	wavelet	Texture Features--GLCM
wavelet_HHH_glcm_JointEntropy	wavelet	Texture Features--GLCM
wavelet_HHH_glcm_Imc1	wavelet	Texture Features--GLCM
wavelet_HHH_glcm_Imc2	wavelet	Texture Features--GLCM
wavelet_HHH_glcm_Idm	wavelet	Texture Features--GLCM

wavelet_HHH_glcm_Idmn	wavelet	Texture Features--GLCM
wavelet_HHH_glcm_Id	wavelet	Texture Features--GLCM
wavelet_HHH_glcm_Idn	wavelet	Texture Features--GLCM
wavelet_HHH_glcm_InverseVariance	wavelet	Texture Features--GLCM
wavelet_HHH_glcm_MaximumProbability	wavelet	Texture Features--GLCM
wavelet_HHH_glcm_SumEntropy	wavelet	Texture Features--GLCM
wavelet_HHH_glcm_SumSquares	wavelet	Texture Features--GLCM
wavelet_HHH_glrlm_GrayLevelNonUniformity	wavelet	Texture Features--GLRLM
wavelet_HHH_glrlm_GrayLevelNonUniformityNormalized	wavelet	Texture Features--GLRLM
wavelet_HHH_glrlm_GrayLevelVariance	wavelet	Texture Features--GLRLM
wavelet_HHH_glrlm_HighGrayLevelRunEmphasis	wavelet	Texture Features--GLRLM
wavelet_HHH_glrlm_LongRunEmphasis	wavelet	Texture Features--GLRLM
wavelet_HHH_glrlm_LongRunHighGrayLevelEmphasis	wavelet	Texture Features--GLRLM

wavelet_HHH_glrlm_LongRunLowGrayLevelEmphasis	wavelet	Texture Features--GLRLM
wavelet_HHH_glrlm_LowGrayLevelRunEmphasis	wavelet	Texture Features--GLRLM
wavelet_HHH_glrlm_RunEntropy	wavelet	Texture Features--GLRLM
wavelet_HHH_glrlm_RunLengthNonUniformity	wavelet	Texture Features--GLRLM
wavelet_HHH_glrlm_RunLengthNonUniformityNormalized	wavelet	Texture Features--GLRLM
wavelet_HHH_glrlm_RunPercentage	wavelet	Texture Features--GLRLM
wavelet_HHH_glrlm_RunVariance	wavelet	Texture Features--GLRLM
wavelet_HHH_glrlm_ShortRunEmphasis	wavelet	Texture Features--GLRLM
wavelet_HHH_glrlm_ShortRunHighGrayLevelEmphasis	wavelet	Texture Features--GLRLM
wavelet_HHH_glrlm_ShortRunLowGrayLevelEmphasis	wavelet	Texture Features--GLRLM
wavelet_HHH_glszm_GrayLevelNonUniformity	wavelet	Texture Features--GLSZM
wavelet_HHH_glszm_GrayLevelNonUniformityNormalized	wavelet	Texture Features--GLSZM

wavelet_HHH_glszm_GrayLevelVariance	wavelet	Texture Features--GLSZM
wavelet_HHH_glszm_HighGrayLevelZoneEmphasis	wavelet	Texture Features--GLSZM
wavelet_HHH_glszm_LargeAreaEmphasis	wavelet	Texture Features--GLSZM
wavelet_HHH_glszm_LargeAreaHighGrayLevelEmphasis	wavelet	Texture Features--GLSZM
wavelet_HHH_glszm_LargeAreaLowGrayLevelEmphasis	wavelet	Texture Features--GLSZM
wavelet_HHH_glszm_LowGrayLevelZoneEmphasis	wavelet	Texture Features--GLSZM
wavelet_HHH_glszm_SizeZoneNonUniformity	wavelet	Texture Features--GLSZM
wavelet_HHH_glszm_SizeZoneNonUniformityNormalized	wavelet	Texture Features--GLSZM
wavelet_HHH_glszm_SmallAreaEmphasis	wavelet	Texture Features--GLSZM
wavelet_HHH_glszm_SmallAreaHighGrayLevelEmphasis	wavelet	Texture Features--GLSZM
wavelet_HHH_glszm_SmallAreaLowGrayLevelEmphasis	wavelet	Texture Features--GLSZM
wavelet_HHH_glszm_ZoneEntropy	wavelet	Texture Features--

		GLSZM
wavelet_HHH_glszm_ZonePercentage	wavelet	Texture Features--GLSZM
wavelet_HHH_glszm_ZoneVariance	wavelet	Texture Features--GLSZM
wavelet_HHH_gldm_DependenceEntropy	wavelet	Texture Features--GLDM
wavelet_HHH_gldm_DependenceNonUniformity	wavelet	Texture Features--GLDM
wavelet_HHH_gldm_DependenceNonUniformityNormalized	wavelet	Texture Features--GLDM
wavelet_HHH_gldm_DependenceVariance	wavelet	Texture Features--GLDM
wavelet_HHH_gldm_GrayLevelNonUniformity	wavelet	Texture Features--GLDM
wavelet_HHH_gldm_GrayLevelVariance	wavelet	Texture Features--GLDM
wavelet_HHH_gldm_HighGrayLevelEmphasis	wavelet	Texture Features--GLDM
wavelet_HHH_gldm_LargeDependenceEmphasis	wavelet	Texture Features--GLDM
wavelet_HHH_gldm_LargeDependenceHighGrayLevelEmphasis	wavelet	Texture Features--GLDM
wavelet_HHH_gldm_LargeDependenceLowGrayLevelEmphasis	wavelet	Texture Features--

		GLDM
wavelet_HHH_gldm_LowGrayLevelEmphasis	wavelet	Texture Features--GLDM
wavelet_HHH_gldm_SmallDependenceEmphasis	wavelet	Texture Features--GLDM
wavelet_HHH_gldm_SmallDependenceHighGrayLevelEmphasis	wavelet	Texture Features--GLDM
wavelet_HHH_gldm_SmallDependenceLowGrayLevelEmphasis	wavelet	Texture Features--GLDM
wavelet_HHH_ngtdm_Busyness	wavelet	Texture Features--NGTDM
wavelet_HHH_ngtdm_Coarseness	wavelet	Texture Features--NGTDM
wavelet_HHH_ngtdm_Complexity	wavelet	Texture Features--NGTDM
wavelet_HHH_ngtdm_Contrast	wavelet	Texture Features--NGTDM
wavelet_HHH_ngtdm_Strength	wavelet	Texture Features--NGTDM
wavelet_LLL_firstorder_10Percentile	wavelet	First Order Features
wavelet_LLL_firstorder_90Percentile	wavelet	First Order Features
wavelet_LLL_firstorder_Energy	wavelet	First Order Features

wavelet_LLL_firstorder_Entropy	wavelet	First Order Features
wavelet_LLL_firstorder_InterquartileRange	wavelet	First Order Features
wavelet_LLL_firstorder_Kurtosis	wavelet	First Order Features
wavelet_LLL_firstorder_Maximum	wavelet	First Order Features
wavelet_LLL_firstorder_MeanAbsoluteDeviation	wavelet	First Order Features
wavelet_LLL_firstorder_Mean	wavelet	First Order Features
wavelet_LLL_firstorder_Median	wavelet	First Order Features
wavelet_LLL_firstorder_Minimum	wavelet	First Order Features
wavelet_LLL_firstorder_Range	wavelet	First Order Features
wavelet_LLL_firstorder_RobustMeanAbsoluteDeviation	wavelet	First Order Features
wavelet_LLL_firstorder_RootMeanSquared	wavelet	First Order Features
wavelet_LLL_firstorder_Skewness	wavelet	First Order Features
wavelet_LLL_firstorder_TotalEnergy	wavelet	First Order Features
wavelet_LLL_firstorder_Uniformity	wavelet	First Order Features
wavelet_LLL_firstorder_Variance	wavelet	First Order Features

wavelet_LLL_glcm_Autocorrelation	wavelet	Texture Features--GLCM
wavelet_LLL_glcm_JointAverage	wavelet	Texture Features--GLCM
wavelet_LLL_glcm_ClusterProminence	wavelet	Texture Features--GLCM
wavelet_LLL_glcm_ClusterShade	wavelet	Texture Features--GLCM
wavelet_LLL_glcm_ClusterTendency	wavelet	Texture Features--GLCM
wavelet_LLL_glcm_Contrast	wavelet	Texture Features--GLCM
wavelet_LLL_glcm_Correlation	wavelet	Texture Features--GLCM
wavelet_LLL_glcm_DifferenceAverage	wavelet	Texture Features--GLCM
wavelet_LLL_glcm_DifferenceEntropy	wavelet	Texture Features--GLCM
wavelet_LLL_glcm_DifferenceVariance	wavelet	Texture Features--GLCM
wavelet_LLL_glcm_JointEnergy	wavelet	Texture Features--GLCM
wavelet_LLL_glcm_JointEntropy	wavelet	Texture Features--GLCM
wavelet_LLL_glcm_Imc1	wavelet	Texture Features--GLCM
wavelet_LLL_glcm_Imc2	wavelet	Texture Features--GLCM
wavelet_LLL_glcm_Idm	wavelet	Texture Features--GLCM

wavelet_LLL_glcm_Idmn	wavelet	Texture Features--GLCM
wavelet_LLL_glcm_Id	wavelet	Texture Features--GLCM
wavelet_LLL_glcm_Idn	wavelet	Texture Features--GLCM
wavelet_LLL_glcm_InverseVariance	wavelet	Texture Features--GLCM
wavelet_LLL_glcm_MaximumProbability	wavelet	Texture Features--GLCM
wavelet_LLL_glcm_SumEntropy	wavelet	Texture Features--GLCM
wavelet_LLL_glcm_SumSquares	wavelet	Texture Features--GLCM
wavelet_LLL_glrlm_GrayLevelNonUniformity	wavelet	Texture Features--GLRLM
wavelet_LLL_glrlm_GrayLevelNonUniformityNormalized	wavelet	Texture Features--GLRLM
wavelet_LLL_glrlm_GrayLevelVariance	wavelet	Texture Features--GLRLM
wavelet_LLL_glrlm_HighGrayLevelRunEmphasis	wavelet	Texture Features--GLRLM
wavelet_LLL_glrlm_LongRunEmphasis	wavelet	Texture Features--GLRLM
wavelet_LLL_glrlm_LongRunHighGrayLevelEmphasis	wavelet	Texture Features--GLRLM
wavelet_LLL_glrlm_LongRunLowGrayLevelEmphasis	wavelet	Texture Features--

		GLRLM
wavelet_LLL_glrlm_LowGrayLevelRunEmphasis	wavelet	Texture Features--GLRLM
wavelet_LLL_glrlm_RunEntropy	wavelet	Texture Features--GLRLM
wavelet_LLL_glrlm_RunLengthNonUniformity	wavelet	Texture Features--GLRLM
wavelet_LLL_glrlm_RunLengthNonUniformityNormalized	wavelet	Texture Features--GLRLM
wavelet_LLL_glrlm_RunPercentage	wavelet	Texture Features--GLRLM
wavelet_LLL_glrlm_RunVariance	wavelet	Texture Features--GLRLM
wavelet_LLL_glrlm_ShortRunEmphasis	wavelet	Texture Features--GLRLM
wavelet_LLL_glrlm_ShortRunHighGrayLevelEmphasis	wavelet	Texture Features--GLRLM
wavelet_LLL_glrlm_ShortRunLowGrayLevelEmphasis	wavelet	Texture Features--GLRLM
wavelet_LLL_glszm_GrayLevelNonUniformity	wavelet	Texture Features--GLSZM
wavelet_LLL_glszm_GrayLevelNonUniformityNormalized	wavelet	Texture Features--GLSZM

wavelet_LLL_glszm_GrayLevelVariance	wavelet	Texture Features--GLSZM
wavelet_LLL_glszm_HighGrayLevelZoneEmphasis	wavelet	Texture Features--GLSZM
wavelet_LLL_glszm_LargeAreaEmphasis	wavelet	Texture Features--GLSZM
wavelet_LLL_glszm_LargeAreaHighGrayLevelEmphasis	wavelet	Texture Features--GLSZM
wavelet_LLL_glszm_LargeAreaLowGrayLevelEmphasis	wavelet	Texture Features--GLSZM
wavelet_LLL_glszm_LowGrayLevelZoneEmphasis	wavelet	Texture Features--GLSZM
wavelet_LLL_glszm_SizeZoneNonUniformity	wavelet	Texture Features--GLSZM
wavelet_LLL_glszm_SizeZoneNonUniformityNormalized	wavelet	Texture Features--GLSZM
wavelet_LLL_glszm_SmallAreaEmphasis	wavelet	Texture Features--GLSZM
wavelet_LLL_glszm_SmallAreaHighGrayLevelEmphasis	wavelet	Texture Features--GLSZM
wavelet_LLL_glszm_SmallAreaLowGrayLevelEmphasis	wavelet	Texture Features--GLSZM
wavelet_LLL_glszm_ZoneEntropy	wavelet	Texture Features--GLSZM

wavelet_LLL_glszm_ZonePercentage	wavelet	Texture Features--GLSZM
wavelet_LLL_glszm_ZoneVariance	wavelet	Texture Features--GLSZM
wavelet_LLL_gldm_DependenceEntropy	wavelet	Texture Features--GLDM
wavelet_LLL_gldm_DependenceNonUniformity	wavelet	Texture Features--GLDM
wavelet_LLL_gldm_DependenceNonUniformityNormalized	wavelet	Texture Features--GLDM
wavelet_LLL_gldm_DependenceVariance	wavelet	Texture Features--GLDM
wavelet_LLL_gldm_GrayLevelNonUniformity	wavelet	Texture Features--GLDM
wavelet_LLL_gldm_GrayLevelVariance	wavelet	Texture Features--GLDM
wavelet_LLL_gldm_HighGrayLevelEmphasis	wavelet	Texture Features--GLDM
wavelet_LLL_gldm_LargeDependenceEmphasis	wavelet	Texture Features--GLDM
wavelet_LLL_gldm_LargeDependenceHighGrayLevelEmphasis	wavelet	Texture Features--GLDM
wavelet_LLL_gldm_LargeDependenceLowGrayLevelEmphasis	wavelet	Texture Features--

		GLDM
wavelet_LLL_gldm_LowGrayLevelEmphasis	wavelet	Texture Features--GLDM
wavelet_LLL_gldm_SmallDependenceEmphasis	wavelet	Texture Features--GLDM
wavelet_LLL_gldm_SmallDependenceHighGrayLevelEmphasis	wavelet	Texture Features--GLDM
wavelet_LLL_gldm_SmallDependenceLowGrayLevelEmphasis	wavelet	Texture Features--GLDM
wavelet_LLL_ngtdm_Busyness	wavelet	Texture Features--NGTDM
wavelet_LLL_ngtdm_Coarseness	wavelet	Texture Features--NGTDM
wavelet_LLL_ngtdm_Complexity	wavelet	Texture Features--NGTDM
wavelet_LLL_ngtdm_Contrast	wavelet	Texture Features--NGTDM
wavelet_LLL_ngtdm_Strength	wavelet	Texture Features--NGTDM

Supplementary Table 2 The selected features and the values of their respective LASSO coefficients

**feature**	**coefficients**
original_shape_Maximum2DDiameterRow	0.007400704
original_shape_Maximum2DDiameterSlice	0.006980187

original_firstorder_Median	2.62156E-05
log.sigma.1.0.mm.3D_gldm_DependenceVariance	0.003408999
wavelet.LLL_firstorder_Median	0.00107798
wavelet.LLL_firstorder_RootMeanSquared	-0.000712108
wavelet.LLL_firstorder_Skewness	-0.070730226
wavelet.LLL_glcm_Idm	-3.317270407
wavelet.LLL_glcm_InverseVariance	1.906724568
wavelet.LLL_glrlm_RunLengthNonUniformityNormalized	-2.688674551
wavelet.LLL_gldm_DependenceVariance	0.02500966

3 Supplementary Figures

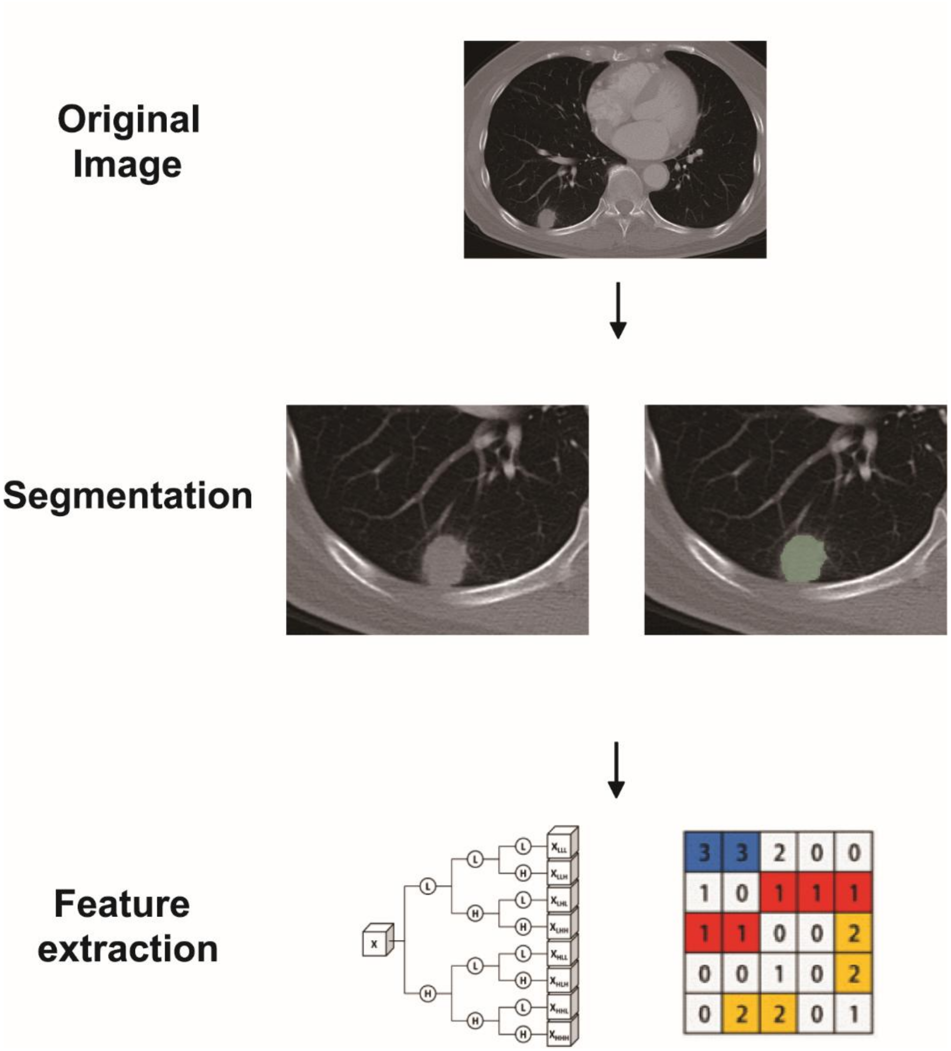

Supplementary Figure 1.
The Radiomics workflow of the study.

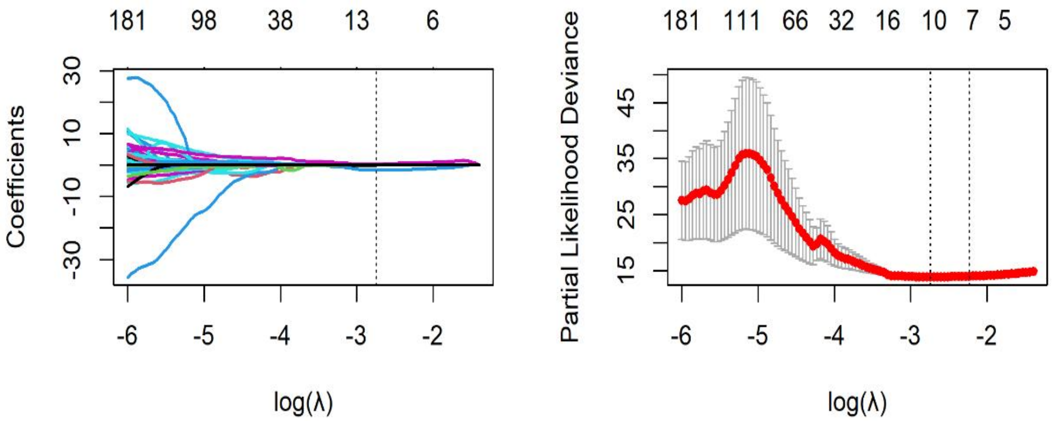

Supplementary Figure 2.
Radiomics feature selection using the LASSO Cox regression model. (Left), each curve represents the change trajectory of each radiomics feature's coefficient distribution. The left column is the value of the coefficients, the lower row is log(λ), and the upper row is the number of non-zero coefficients in the model. Dotted vertical lines represents the optimized hyperparameter λ by 10-fold cross-validation, which ensured that the model had the minimum deviance. The optimal λ is 0.064. (Right) Partial likelihood deviance was generated against the log (λ) sequence. Left dotted vertical line represents the optimal λ values by using the 1 standard error of the minimum criteria (the 1-SE criteria), while right dotted vertical line was drawn using the minimum criteria that minimizes the partial log-likelihood deviance.

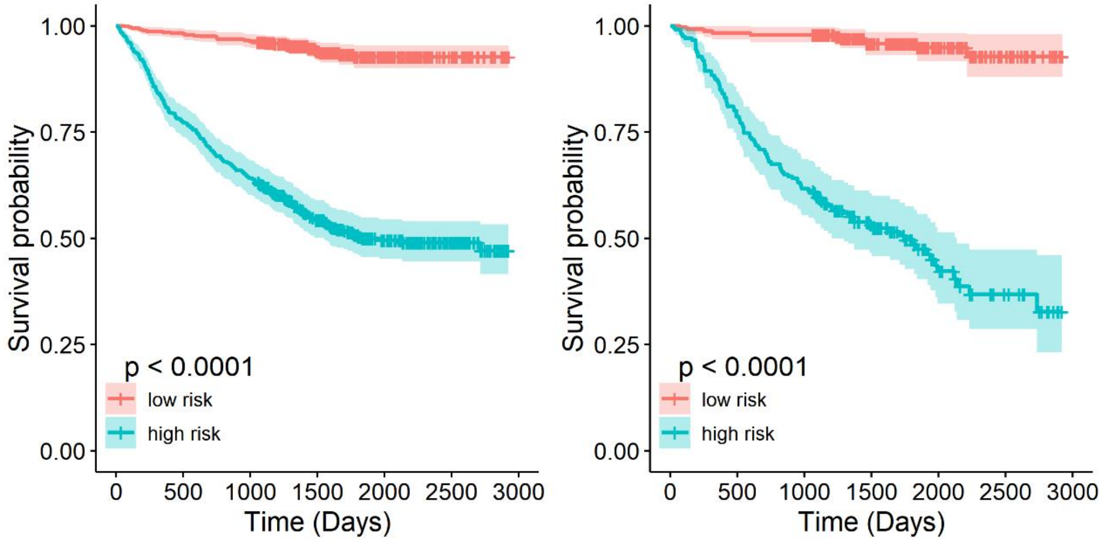

Supplementary Figure 3.
Kaplan-Meier survival analysis of the patients in the high-risk and low-risk groups in the training cohorts (left) and validation cohorts (right). The median of the radiomics signature in the training cohort was used to stratify patients into low-risk group (radiomics signature <= -0.716) and high-risk group (radiomics signature > -0.716) in the two cohorts. A significant difference in the overall survival was found between the two cohorts (*P*<0.001).

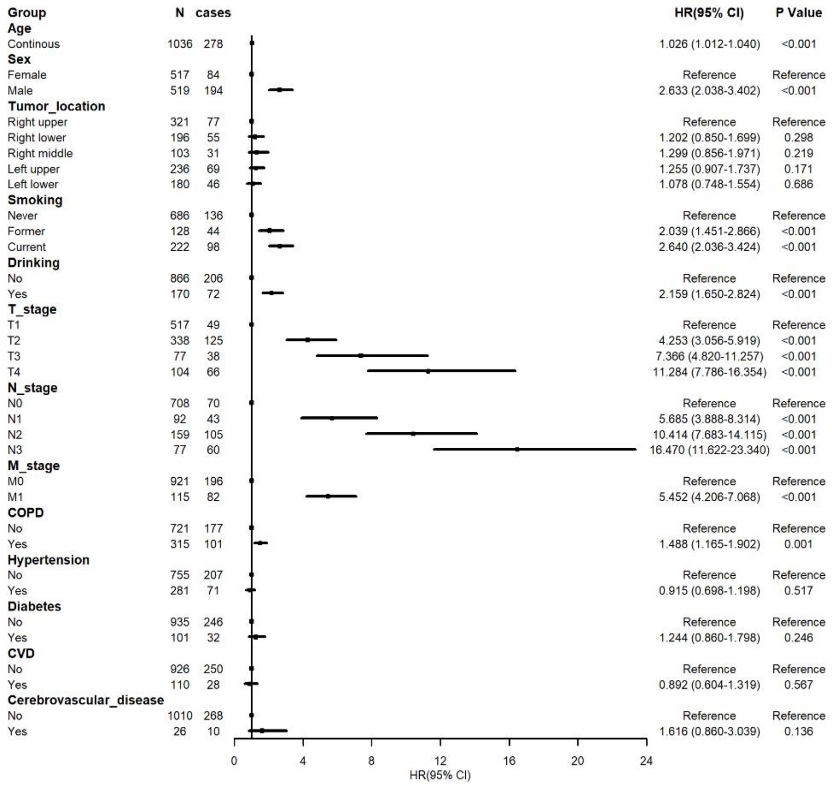

Supplementary Figure 4.
Forest plot with hazard ratios showing univariate Cox regression analyses estimated for association between the clinical variables and overall survival of patients with NSCLC in training cohort.

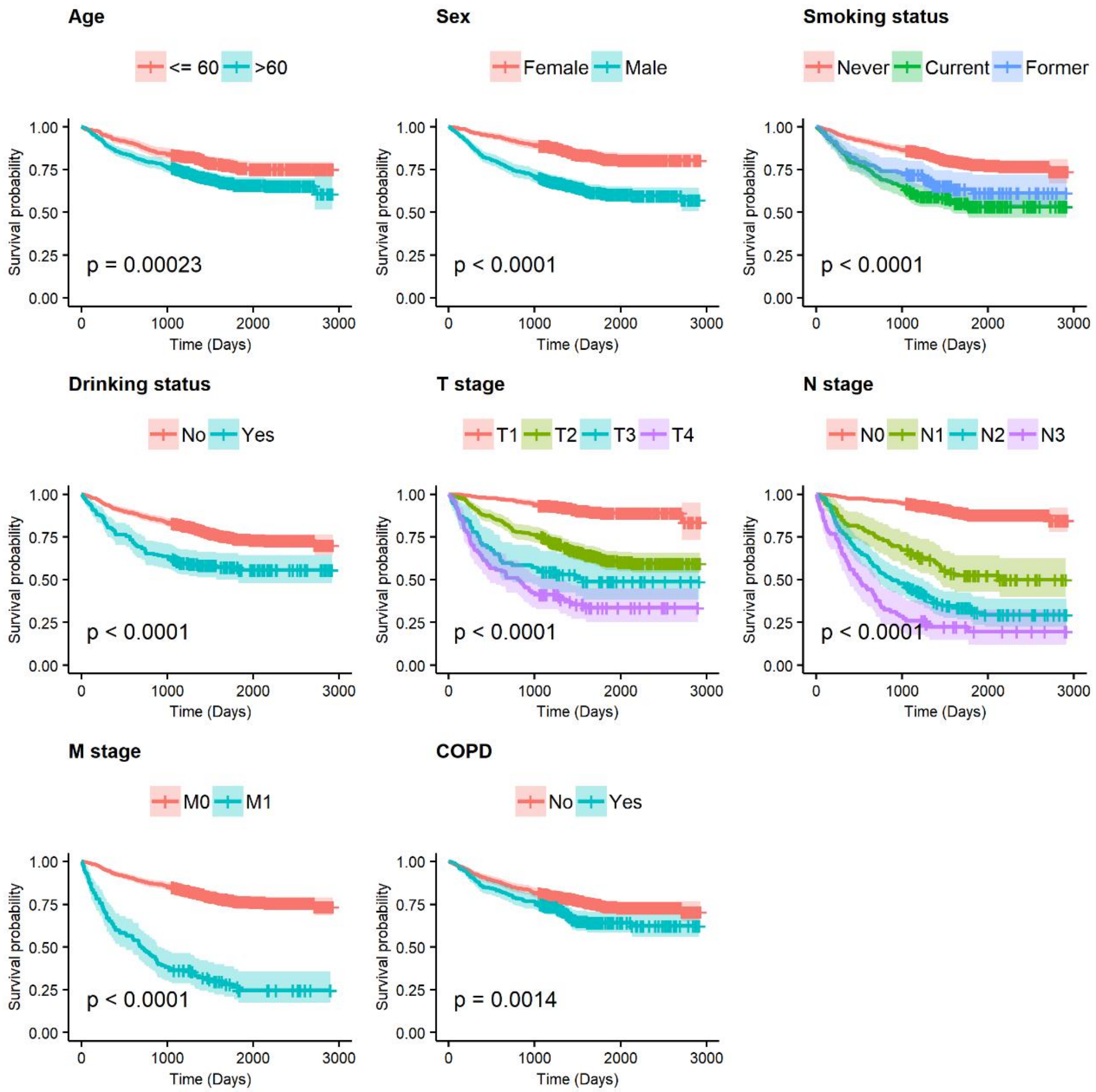

Supplementary Figure 5.
Kaplan-Meier survival analysis showed significant difference in the overall survival by each clinical factor with a *P* value < 0.01 at univariable analysis in the training cohort.

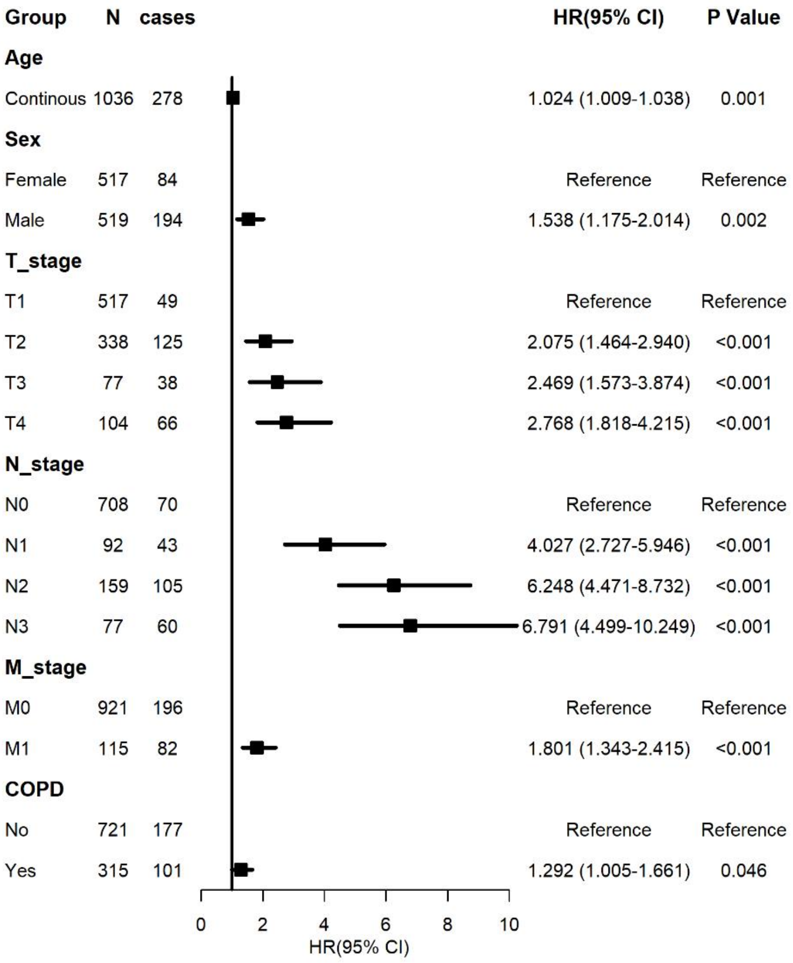

Supplementary Figure 6.
Forest plot with hazard ratios showing multivariate Cox regression analyses estimated for association between the clinical variables and the overall survival of patients with NSCLC in training cohort.

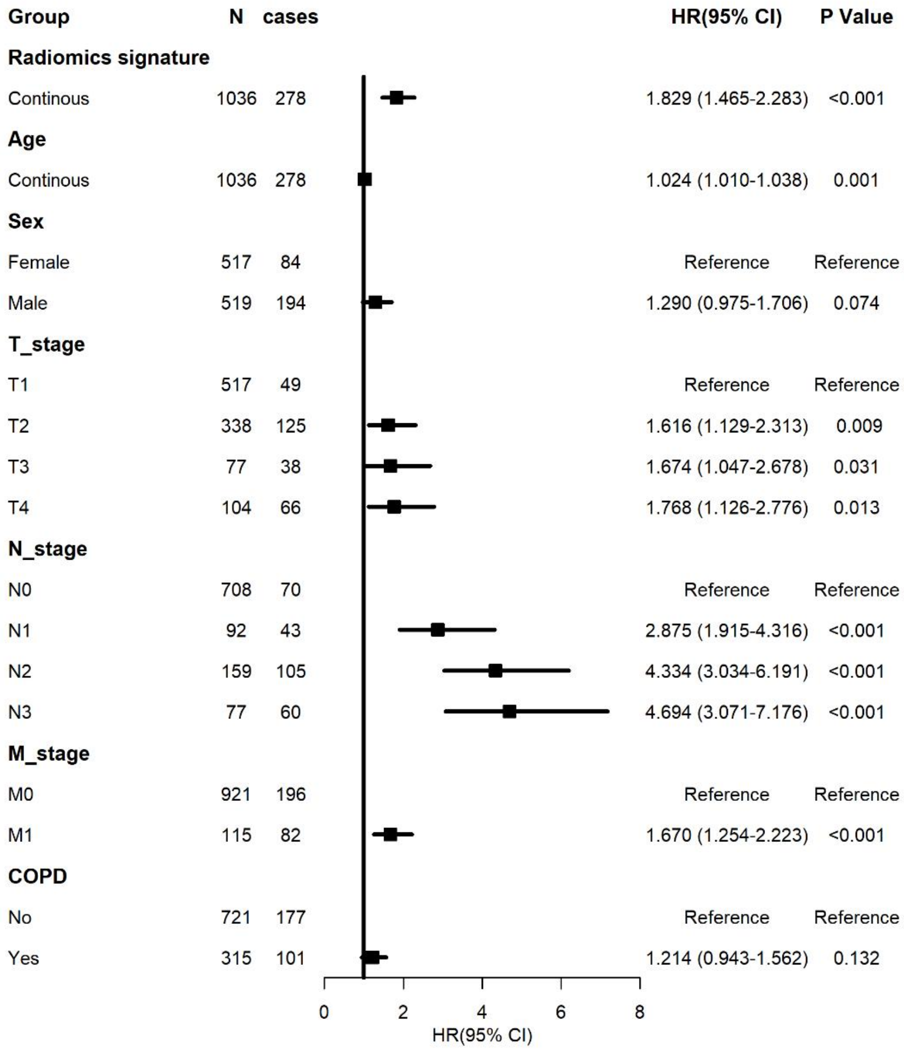

Supplementary Figure 7.
Forest plot with hazard ratios showing multivariate Cox regression analyses estimated for association between the clinical variables and the radiomics signature and overall survival of patients with NSCLC in training cohort.

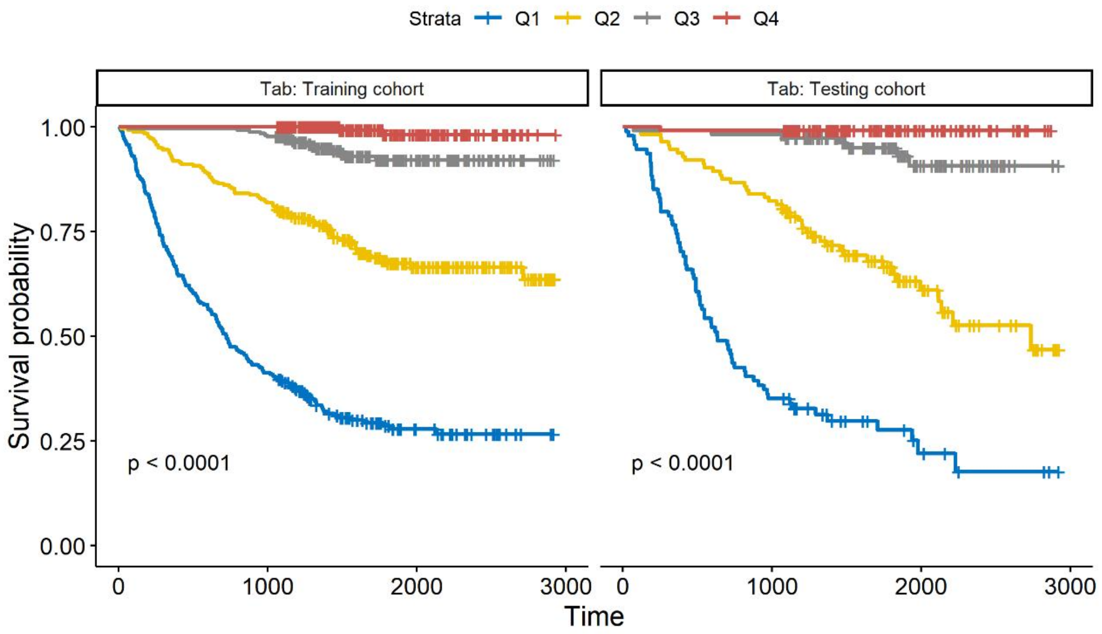

Supplementary Figure 8.
Survival curves of quartiles based on 3-year nomogram predicted risk in training cohort (left) and validation cohort (right).
